# Medical Brain Drain: The Role of Job Attributes and Individual Traits

**DOI:** 10.1002/hec.70148

**Published:** 2026-08-24

**Authors:** Marco Bertoni, Debdeep Chattopadhyay, Yuanyuan Gu

**Affiliations:** ^1^ University of Padova GLO HEDG IZA Netspar Padova Italy; ^2^ Open Health Group Rotterdam the Netherlands; ^3^ Macquarie University Centre for the Health Economy Macquarie University Sydney Australia

**Keywords:** brain drain, job design, medical workforce, selective migration

## Abstract

Medical brain drain poses a major challenge for health systems, reducing physician supply and undermining public investment in medical training. Prior work examined migration flows and their macroeconomic determinants. This study uses a discrete choice experiment to assess how individual background and personality traits shape medical student intentions to migrate and whether job attributes are valued differently at home and abroad. We surveyed 527 senior medical students from 12 Italian universities between November 2020 and July 2021, using Italy as a pertinent case given high physician emigration and limited inflow of foreign‐trained doctors. We find that students require compensation of around 30% of physicians' average starting salary to accept a job abroad rather than at home. Those from higher‐quality schools or medical families, and those who are more altruistic, competitive, and risk‐tolerant, require less compensation to migrate. Valuations of several job attributes also differ by location: at home, students value permanent contracts and professional development; abroad, they are willing to accept temporary contracts but reject under‐skilling. These findings suggest that emigration may disproportionately involve the best‐trained students. Policy simulations show that improving working conditions, job security, or professional development opportunities each substantially increases the predicted probability of choosing a domestic job, with salary increases having the largest effects.

## 1 Introduction

Modern labor markets are increasingly shaped by international job searches, particularly in occupations facing global shortages such as engineering, information technology, and medicine (Arjan et al. 2022). Among these, medical doctors deserve particular attention. Unless countries succeed in attracting skilled physicians from abroad or facilitating the return of migrating natives, medical brain drain may undermine healthcare quality and population health. Moreover, as medical training is very costly and often subsidized by governments, medical emigration generates additional fiscal concerns (Bhagwati and Hamada 1974).

The literature on medical migration has focused on quantifying migration flows, identifying macro‐level determinants, and assessing consequences for sending countries, particularly in low‐income contexts (Clemens and Pettersson Gelander 2006; Bhargava and Docquier 2008; Bhargava et al. 2011). Results emphasize the role of income differentials, institutional quality, and immigration policies. For instance, Adovor et al. (2021) show that economic characteristics at source and destination countries, linguistic and geographical proximity, and lax immigration regimes are drivers of medical migration flows.

Our analysis, by contrast, shifts the focus from macro‐level flows to the micro‐level determinants of medical migration. We contribute to the literature in two complementary ways. First, we uncover the variation in the migration intentions of medical students. While a growing body of evidence documents the role of individual characteristics, cognitive and non‐cognitive traits in triggering migration intentions in the general population (Jaeger et al. 2010; Hao et al. 2016; Fouarge et al. 2019; Bütikofer and Peri 2021; Nejad and Schurer 2022), less is known about medical students—the group that represents the future medical workforce. Understanding who is more likely to stay or leave is crucial, as selective migration may affect not only the size but also the composition and quality of the physician pool in the home country.

Second, we extend the literature on the value of job amenities by examining whether medical student evaluations of job attributes depend on job location. The theory of compensating differentials highlights that workers trade off wages with non‐monetary job attributes, and a wide body of evidence showing that such preferences vary across groups and predict actual job choices in the general population (Mas and Pallais 2017; Wiswall and Zafar 2018; Datta 2019; Le Barbanchon et al. 2021; Feld et al. 2022; Non et al. 2022; Maestas et al. 2023). Several contributions apply Discrete Choice Experiments (DCEs) to health professionals, investigating preferences for job attributes in different settings. Notable examples include Scott (2001), who designed a DCE to examine GP's preferences for job characteristics when choosing a general practice in which to work, and Sivey et al. (2012), who used a DCE to assess what job characteristics are most important to attract medical students to certain specialties. Moreover, Kolstad (2011) and Holte et al. (2015) adopted DCEs to study the attraction and retention of medical workforce in rural areas. Yet, no study has used a DCE to analyze the international migration intentions of medical students and whether they attach different values to given characteristics of jobs located at home and abroad. Addressing these gaps is essential to understand selection into migration and identify job features that can help retain mobile medical students in their home country.^1^

As we are not aware of real‐world experiments collecting information on the job choices of medical students faced with random offers for jobs with different attributes and located in different countries, we designed a novel DCE. In each task, respondents chose between two hypothetical jobs differing in several attributes or opted out, thereby trading off job characteristics in a controlled setting.

A distinctive feature of our design is that job location was included as one of the attributes. This allows us to estimate how much students value working in their home country relative to abroad, and to examine whether the valuations of other job features—professional development opportunities, job security, working conditions, and skill mismatch—vary with job location. In addition, the DCE was complemented with a rich survey on socio‐demographic background, motivations, and cognitive and non‐cognitive traits, enabling us to analyze how preferences interact with individual characteristics.

We implemented the survey between November 2020 and July 2021 among medical students from 12 Italian universities. Italy represents a very relevant case to analyze. It is a European country that has long been affected by the brain drain (Hellemans 2001; Becker et al. 2004), and medical emigration poses a serious challenge to the sustainability of its healthcare system. Around 1000 doctors—roughly one in seven new specialists—leave the country every year to work abroad (Riccò et al. 2020), and Italy ranked fourth in Europe for medical emigration between 1990 and 2014 (Adovor et al. 2021). At the same time, the number of foreign‐trained doctors practicing in Italy is far below the OECD average (OECD 2021), further aggravating shortages (La Colla 2019).

Although participation in the survey was voluntary, we ensured representativeness by reweighting the data through entropy balancing on age, gender, and macro‐region (Hainmueller 2012). We analyzed the data using mixed logit models estimated in the Willingness to Pay (WTP) space (Train and Weeks 2005), which allowed us to directly quantify trade‐offs between wages and other job attributes.

By combining evidence on individual selection and on the valuation of job attributes, our study sheds light on the drivers of medical migration from a high‐income country. The findings have direct implications for policy, as they identify the types of contracts and working conditions that can help retain talented young doctors domestically and mitigate the challenges posed by the medical brain drain—a challenge faced not only by Italy, but by many healthcare systems worldwide.

## 2 Survey Design

### 2.1 The Discrete Choice Experiment

A DCE is a stated preference method that elicits individuals' preferences by presenting them with alternatives defined by a set of attributes. Researchers generate unique alternatives by varying the levels of these attributes and then ask respondents to make repeated choices between two or more options in a series of scenarios. By analyzing the resulting choice patterns, researchers can identify the relative importance of the attributes and the trade‐offs respondents are willing to make. Typically, one attribute is expressed as a continuous monetary variable, which enables preferences for other attributes to be translated into willingness to pay.

In our DCE, we ask senior medical students to assume that they had just completed their specialization of choice, and confront them with 16 choice tasks. In each task, they choose between two hypothetical jobs that differ with respect to several attributes.^2^ We also included an opt‐out option, and the selection of this option signifies that neither of the job offers meets the student's reservation level of utility. Because in reality job seekers are rarely forced to choose between only two offers, forcing the choice of a job among the proposed ones could have generated unrealistic choice patterns. According to Almalaurea data,^3^ the unemployment rate of Italian medical graduates at 5 years from graduation is almost negligible, below 1%, compared to an average of 7% among all other graduates. This means they do not have to worry too much about unemployment risk when making choices, and it is realistic to think that they may turn down offers that don't meet their reservation utility. In the data, we observe that respondents chose opt‐out 11.9% of the times in total. At the same time, no respondent opted out all times.

The selection of attributes was informed by both the existing literature on physicians' job preferences (see e.g. Mas and Pallais 2017; Datta 2019; Non et al. 2022; Maestas et al. 2023; Kolstad 2011; Holte et al. 2015) and a focus group conducted with senior medical students at the University of Padova. The focus group discussion confirmed the relevance of the attributes identified in the literature and helped refine their interpretation and wording in the survey instrument. While participants also highlighted that an initial career decision concerns the broader type of career (e.g., clinical work vs. alternative career paths), the discrete choice experiment was restricted to hospital doctor positions in order to keep the job attributes comparable across alternatives. A detailed description of the attribute development process and a summary of the focus group discussion are provided in Supporting Information S1: Online Appendix E. The eventual choice of attributes and their corresponding levels is reported in Table 1 and described in what follows. Supporting Information S1: Online Appendix Figure A1 reports the introduction to the choice tasks provided to students and a choice task example.

1 *Location.* In the survey, students were initially asked to name their favorite foreign European country. We restricted the choice set to European countries to limit hypothetical bias.^4^ When thinking about their favorite foreign European country, we prompted students not to consider aspects related with the labor market—such as wages, working conditions, career prospects, training or research opportunities—but rather factors that attract them independently of work—such as weather, food, culture, or the functioning of institutions. Supporting Information S1: Online Appendix Table A1 reports the distribution of the favorite European country selected by individuals in our final sample, and shows that the UK, Spain, Switzerland and France are the most commonly chosen countries. Geographic and cultural proximity, as well as language familiarity, help to explain these patterns. This ranking closely mirrors OECD data on the emigration of Italian doctors in 2000–2021, where the UK, Switzerland, and France also rank among the top five destinations, together with Germany and Belgium.
  We then presented students with jobs located either in Italy or in their favorite foreign European country. We chose this approach for two reasons. First, referring simply to a job “abroad” could have introduced unobserved heterogeneity in WTP estimates, as students might interpret “abroad” in different ways. Second, naming a specific foreign country would have created the opposite problem, because students hold different perceptions of particular countries.^5^ Moreover, to ease the identification of preferences for other job attributes, the design required students to make comparisons not only between jobs located in different countries but also between jobs located in the same country (Italy or their favorite foreign European country).^6^
2. *Income.* Income served as the numeraire variable, allowing us to calculate willingness‐to‐pay (WTP) for changes in the levels of all other attributes. In other words, we estimated how much income a student was willing to sacrifice to move from one level of an attribute to a more preferred one. We presented students with Purchasing Power Parity (PPP)‐adjusted net income levels. To account for differences in the cost of living, we instructed them that if a job was located in a country where the cost of living was higher (lower) than in Italy, they should assume proportionally higher (lower) earnings, so that their purchasing power would be equivalent to what the same income would provide in Italy. Because this instruction referred to the overall cost of living, it covered all aspects of life—including housing, schooling, and healthcare—thus reducing potential confounding from heterogeneity in housing costs or social security coverage across countries. We also asked students to assume that the stated salary applied to the first 2 years of the job, and would increase by 5% every 2 years (see Anelli 2019). The choice of income levels was based on OECD (2019) data on doctors' salaries in European countries, and the details of how we set these levels are reported in Supporting Information S1: Online Appendix B.
3. *Job security.* This attribute represents the duration of the contract, and can be expected to influence doctors' choices not only by itself but also through an interaction effect with the country of work. For example, if an individual wants to work abroad only for a few years to enjoy the experience of working and living abroad, but wants to settle in Italy in the long term, then obtaining a permanent job abroad would not be as important as it might be in the home country.
4. *Professional development opportunities.* This attribute refers broadly to the possibility of engaging in training and research activities, as opposed to performing purely routine clinical work, during the early stages of the career. The impact of this attribute on choice may differ by job location. If migration intentions are only temporary, professional development opportunities may be perceived to be more valuable for jobs located in the home country, where medical students want to settle in the long run.
5. *Working conditions.* Working conditions or workload is a highly relevant attribute considered by doctors making a job choice and has been used by many studies on specialty choice and medical staff preferences (Kolstad 2011; Sivey et al. 2012). Their impact may differ by job location if doctors are willing to stand bad working conditions for a short period abroad in exchange for the “foreign” experience.
6. *Match of skills with job content.* Skill mismatch is a relevant determinant of job turnover and job satisfaction, as well as of individual productivity at work (see Brunello and Wruuck 2021, for a review). While over‐skilling may generate frustration, under‐skilling requires training. The impact of this attribute on choice may also differ by job location. For example, individuals may be unwilling to invest in skills for a job abroad if they already face other non‐monetary costs related with adaptation to a foreign environment—including language training.

**TABLE 1:** Job attributes and their levels.

Attribute	Levels
Location	Italy
	Your favorite european foreign country
Annual net income (PPP adjusted)	€20.000
	€30.000
	€40.000
	€50.000
	€60.000
	€75.000
Job security	2‐year fixed term contract, non‐renewable
	2‐year fixed term contract with 50% renewal probability
	Permanent job
Professional development opportunities	Limited opportunities for research and training
	Some opportunities for research and training
	Good opportunities for research and training
Working conditions	High workload with frequent overtime work and night shifts
	Adequate workload with little overtime work and night shifts
Match of skills with job content	Your skills are higher than required by the job
	Your skills are exactly matched to what is required by the job
	Some of your skills are lower than required by the job
	and need further development

A full factorial design using these six attributes and their defined levels would lead to $\left(2^{2}\times 3^{3}\times 6\right)=648$ different job profiles and ^648^ $C_{2}$ =209,628 possible choices between any two jobs. Since this is clearly not feasible to be implemented, we used a D‐efficient design to restrict the number of choice sets to 16. We obtained the design by specifying the most complex version of the model that we were interested in estimating, which included up to a cubic income term and interaction terms between country and other attributes. In addition to the 16 tasks selected with the D‐efficient procedure, we included an additional task in which one job option strictly dominated the other. This serves as a check for whether subjects were paying sufficient attention while participating in the experiment and/or whether they understood the instructions provided to complete the DCE tasks. We excluded from the final sample 5 subjects who did not select the strictly dominant job option (see Section 3.2). To reduce the potential influence of attribute presentation order, respondents were randomly assigned to one of three questionnaire versions, each presenting the job attributes in a different vertical order. Within each questionnaire version, the attribute order was fixed across choice tasks. We adopted this approach to reduce cognitive burden by allowing respondents to compare the same attributes in the same row positions throughout the DCE, while still varying attribute order across respondents.

### 2.2 Background Characteristics and Personality Traits

After finishing the choice tasks, we asked respondents to complete a comprehensive questionnaire on their socio‐demographic characteristics and their personality traits. Background information includes the students' age, gender, country and region of birth, their specialization area of interest and if they'd like to undertake the specialization in the same University where they are currently enrolled, their grade point average at university, whether they have a partner, whether they participated in an Erasmus exchange program, family income and parents' occupations, whether they know the language of their favorite European country, and whether any of their personal ties lives there.

We have also collected various personality characteristics of the students, that include self‐efficacy (individuals' belief in their capacity to undertake given tasks); locus of control (the degree to which people believe that the control over the outcome of events in their lives depends on themselves or on external forces); intrinsic and extrinsic motivation (whether people perform tasks for the pleasure of doing them or because of outside causes, such as punishments or rewards); risk attitudes; competitiveness; altruism; and the Big‐5 personality traits (openness, conscientiousness, extraversion, agreeableness, neuroticism).

References and details for the survey items used for the measurement of these traits and the construction of the corresponding indices are reported in Supporting Information S1: Online Appendix C. In order to understand selection into migration, in the empirical analysis we will investigate the heterogeneity in medical students' WTP for remaining in Italy along these traits.

## 3 Data

### 3.1 Sampling

We conducted an online survey (in Italian) with medical students enrolled in Italian universities. We refer readers to Supporting Information S1: Online Appendix D for a detailed presentation of the institutional setup governing medical education and access to the medical profession in Italy. To gain permission to contact the students and to reach them with appropriate communication strategies, we got in touch with all the Italian universities with a medical school via the National Board of Medical School Deans, and by writing individual emails to all deans and course managers. In total, 12 out of 40 medical schools replied to our appeal and supported us in the data collection (11 are public, one is private). Supporting Information S1: Online Appendix Table A2 reports the list of universities involved in our study and the distribution of respondents in our final sample by university.

We made the survey instrument available to students through a dedicated online link. We included the link in an invitation message and forwarded the text to the deans. Then, each university autonomously decided the most effective way to engage the students in the survey (some acted via the students' representatives, other via students' electronic board, other used emails). We collected the data in November 2020 at the University of Padova, that served as a pilot, and between April and July 2021 at all other Universities. A pilot survey was conducted with 90 medical students at the University of Padova in November 2020. Two respondents failed the dominance check and were excluded, leaving 88 pilot respondents. Among them, all reported the choice tasks as very clear (60 respondents, 68.2%) or clear (28, 31.8%). On a difficulty scale from 1 (not difficult at all) to 3 (very difficult), 36 respondents (40.9%) rated the tasks as not difficult at all, 51 (58.0%) as difficult, and 1 (1.1%) as very difficult, indicating that the cognitive burden was manageable. The median completion time was 22 minutes (P10–P90: 14–37 minutes), and the pilot responses did not indicate increasing opt‐out rates across tasks, suggesting limited fatigue or disengagement effects. As our experiment requires the students to imagine themselves after their specialization, we follow Non et al. (2022) and minimize hypothetical bias by only selecting students enrolled in the fourth to final (sixth) grade—who already had clinical experience in hospital wards (see Supporting Information S1: Online Appendix D)—and who stated that they are planning to attend a specialization course.

### 3.2 Sample Selection

We collected data on 544 medical students enrolled in grades 4–6 who aim to pursue a specialization—the others were automatically excluded from completing the DCE task by the survey instrument. From this sample, we dropped 6 non‐Italian students, 2 students who listed a non‐European favorite country, 5 students who ticked the strictly‐dominated job offer, thereby failing the rationality test, 4 students with missing data on key covariates. Our final sample includes 527 respondents, each completing 16 choice tasks, for a total of 8432 choices.

### 3.3 Sample Size Considerations

We did not conduct a formal ex ante power calculation. Formal power calculations for DCEs are challenging because power depends on the experimental design, the number of choice tasks, the number and balance of attribute levels, assumed preference parameters, model specification, error variance, and the distribution of respondents across subgroups. This is particularly true for mixed logit models and heterogeneity analyses, for which there is no single universally accepted analytical formula equivalent to a conventional power calculation for a simple mean difference or regression coefficient.

As a benchmark for the main DCE model, we considered the commonly used Johnson and Orme rule‐of‐thumb for choice‐based conjoint/DCE studies (Johnson and Orme 2003; Orme 2010; de Bekker‐Grob et al. 2015):

$$N\geq \frac{500c}{t\times a}$$

where c is the largest number of levels for any attribute, t is the number of choice tasks completed by each respondent, and a is the number of alternatives per choice task. In our design, the largest number of levels for an attribute was six, respondents completed 16 experimental choice tasks, and each task included two job alternatives plus an opt‐out alternative. Counting the opt‐out alternative gives a=3, implying a minimum benchmark sample size of approximately 63 respondents. If only the two job alternatives are counted, the more conservative benchmark is approximately 94 respondents. Our final sample of 527 respondents, providing 8432 observed choices, is therefore well above both benchmarks for estimating the baseline DCE model. However, this rule‐of‐thumb should be interpreted only as a broad benchmark, and not as a formal power calculation for the mixed logit model or for subgroup analyses.

### 3.4 Sample Representativeness

A relevant issue in terms of the external validity of our findings concerns sample representativeness. While all targeted students at each participating university were invited to take the survey, not all of them did so. This non‐response problem may lead us to analyze a sample that potentially does not represent the reference population. To assess the representativeness of our sample and correct for differential non‐response, we obtained population‐level statistics on age, gender, and macro‐region of medical students enrolled in years 4–6 in the participating universities, using data from the register of university students held by the Italian Ministry of Education. We then applied the minimum entropy balancing method (Hainmueller 2012) to reweight the respondent sample so that it matches these population moments.^7^ The role of the weights is therefore to correct for differential response rates across observable characteristics within the relevant population of medical students, rather than to reweight the sample to the broader population of Italian university students. Supporting Information S1: Online Appendix Table A3 shows how the original and the reweighted sample compare to the target population statistics. We conclude that reweighting attributed relatively less weight to respondents from Northern Italy, that are over‐represented in the sample. Descriptive statistics on the socio‐demographic characteristics of our sample are presented in Supporting Information S1: Online Appendix Table A4. The Table compares the mean values of many variables of interest before and after reweighting. Eventually, the impact of reweighting is marginal.

### 3.5 Choice Task Checks

After completing the choice task, we prompted students with some questions about the choice tasks. Following the literature (e.g., see Lancsar et al. 2020), we asked respondents about their self‐reported difficulty level of understanding and answering to the DCE task. On understandability, all subjects in our final sample reported that the task was either very clear (65%) or clear (35%), and no subject stated that it was unclear or very unclear. On difficulty, 52% of subjects rated the tasks as not difficult, 46% as difficult, and 2% as very difficult. Overall, the results support the acceptability of the task from respondents, and confirm the initial views of the medical student representatives in Padova, who deemed the choice tasks as feasible in a pre‐test phase. We also asked students to rank the listed job attributes according to their self‐assessed importance. We found that the average ranking of self‐assessed attributes' importance mirrors the ranking of attributes by the average WTPs that we estimate from the mixed logit model. Overall, these findings reassure us about the internal consistency of the choices.

Finally, we also directly inquired those students who participated in 2021 about how much they think the Covid‐19 pandemic affected their decision to move abroad. Close to 69% of respondents reported that Covid‐19 had no impact on their migration intention, 20% have a stronger preference to stay, and 11% have a stronger preference to move. If anything, our setup could thus be overestimating how much students would need to be paid to leave their home country in the post‐pandemic era.

## 4 Empirical Methodology

Our analysis is based on the random utility framework (Mcfadden 1973), assuming that individuals maximize expected utility from job alternatives subject to random error. For individual i choosing job j in task t, utility is:

(1) $$U_{\mathrm{ijt}}={ASC}_{j}+\alpha_{i}Income_{jt}+X_{\mathrm{jt}}\beta_{i}+\varepsilon_{\mathrm{ijt}},$$

where i=1,…,N, t=1,…,16, and j=1,2,3 (with j=3 denoting the opt‐out). ${ASC}_{j}$ are alternative‐specific constants capturing order biases in picking jobs located in the left or right column of the screen; $Income_{jt}$ is the income of job j; and $X_{\mathrm{jt}}$ is a vector of non‐monetary attributes (professional development, job security, working conditions, skill match, and country). Coefficients $\alpha_{i}$ and $\beta_{i}$ capture individual‐level preferences, and the error term is assumed extreme‐value distributed. Under standard assumptions, choice probabilities are estimated using a mixed logit model (Mcfadden and Train 2000).

Preferences can also be expressed as willingness‐to‐pay (WTP) for non‐monetary attributes, defined as the marginal rate of substitution between attribute a and income:

(2) $$WTP_{i}^{a}=\frac{\beta_{i,a}}{\alpha_{i}}.$$

Distributional assumptions on coefficients determine the distribution of WTPs. A common approach fixes $\alpha_{i}$ while allowing $\beta_{i}$ to vary normally, but this imposes homogeneity in income preferences. Allowing $\alpha_{i}$ to follow a log‐normal distribution introduces heterogeneity and ensures a positive income effect, though it can yield skewed WTP distributions (Train and Weeks 2005; Hole and Kolstad 2012).

To address this, we estimate the model in “WTP space” (Hole and Kolstad 2012), rewriting utility as:

(3) $$U_{\mathrm{ijt}}=\alpha_{i}\left[{ASC}_{j}+Income_{jt}+X_{\mathrm{jt}}{WTP}_{i}\right]+\varepsilon_{\mathrm{ijt}}.$$

We assume normal distributions for WTPs and a log‐normal distribution for $\alpha_{i}$. Estimation uses simulated maximum likelihood with clustered standard errors at the individual level.^8^

To characterize heterogeneity in migration intentions, we examine how medical students' WTP for job location varies with observable traits $T_{i}$, defined as dummy variables. For example, $T_{i}=1$ if individual i is female and 0 otherwise. We model utility as:

(4) $$\begin{array}{l} U_{\mathrm{ijt}}=\alpha_{i}\left[{ASC}_{j}+Income_{jt}+{\tilde{X}}_{jt}{\tilde{WTP}}_{i}\right.\\ \;\left.+WTP_{i}^{c0}Country\times \left(1-T_{i}\right)+WTP_{i}^{c1}Country\times T_{i}\right]+\varepsilon_{\mathrm{ijt}}, \end{array}$$

where $WTP_{i}^{c0}$ and $WTP_{i}^{c1}$ denote the distributions of WTP for job location across groups defined by $T_{i}$. For continuous traits, we split the sample at the median. While the income parameter $\alpha_{i}$ is restricted to have the same distribution across groups, we consider this acceptable given that income is expressed in PPP terms and respondents were explicitly reminded of this interpretation. Compared with estimating in separate sub‐samples, the interaction specification facilitates hypothesis testing on whether WTP distributions differ significantly across groups. Furthermore, since traits are not randomly assigned, these results should be interpreted descriptively.

Another part of the analysis investigates whether WTP for non‐monetary job attributes differs by job location. We decompose $X_{\mathrm{jt}}=\left(Country_{jt},{\tilde{X}}_{jt}\right)$, with $Country_{jt}=0$ for Italy and 1 for abroad. For each attribute ${\tilde{X}}_{jt}^{a}\in {\tilde{X}}_{jt}$, we extend Equation (3) by including an interaction term between $Country_{\mathrm{ijt}}$ and ${\tilde{X}}_{jt}^{a}$:

(5) $$U_{\mathrm{ijt}}=\alpha_{i}\left[{ASC}_{j}+Income_{jt}+X_{\mathrm{jt}}{WTP}_{i}+Country_{jt}\times {\tilde{X}}_{jt}^{a}WTP_{i}^{ca}\right]+\varepsilon_{\mathrm{ijt}},$$

where $WTP_{i}^{ca}$ measures the additional willingness to pay for attribute ${\tilde{X}}_{jt}^{a}$ when the job is abroad. We estimate this separately for each attribute.

To translate the estimated preferences into policy‐relevant quantities, we also use the baseline mixed logit model to simulate predicted choice probabilities under selected job‐offer scenarios. The simulations compare an Italian job with a job in the respondent's most desirable country in the rest of Europe. For each scenario, we define the attribute levels of the two job offers and calculate the mean predicted probability of choosing the Italian or foreign job. The simulations are based on the baseline model because it includes all job attributes simultaneously and therefore provides internally consistent predictions for complete job bundles. The predicted probabilities should be interpreted as conditional choice probabilities between the two job offers within the experimental setting, not as direct forecasts of aggregate migration flows or actual retention rates.

## 5 Results

### 5.1 Job Location Preferences

Table 2 shows the WTPs estimated from the mixed logit model outlined in Equation (3). Column (1) reports the estimated means of the distribution of job attributes' WTPs, and Column (2) reports the estimated SDs.

**TABLE 2:** Baseline WTP‐space mixed logit estimates. Mean and SD.

Variable	Mean	SD
Job location WTP—reference: Italy		
Favourite foreign EUR country	−13,515***	17,216***
	(1298)	(1501)
Professional development opportunities WTP—reference: poor dev. opp.		
Some dev. opp.	6572***	8182***
	(947)	(1122)
Good dev. opp.	10,998***	7641***
	(941)	(934)
Job security WTP—reference: fix‐term, non‐renewable job		
Fixed‐term job, 50% renew prob.	11,800***	4920***
	(838)	(1185)
Permanent job	8878***	13,071***
	(1009)	(971)
Working conditions WTP—reference level: poor		
Good working conditions	12,740***	8408***
	(830)	(996)
Match of skills with job content WTP—reference: higher than needed		
Exactly matched	3711***	5809***
	(905)	(1539)
Lower than needed	3002***	8849***
	(925)	(919)
Income $\left(\alpha_{i}\right)$	0.126***	0.106***
	(0.009)	(0.008)
$ASC_{1}$	−37,686***	5115***
	(1658)	(791)
$ASC_{2}$	−39,961***	2931***
	(1761)	(854)
Choices	8432	
Subjects	527	

*Note:* This table reports estimates obtained from Equation (3). $ASC_{1}$ and $ASC_{2}$ are alternative‐specific constants for the first and second job offers, capturing position effects (i.e., systematic tendencies to prefer the left or right alternative irrespective of its attributes). Observations are weighted to match the population level statistics of the share of females, age and share of students by macro‐regions. Robust standard errors in parentheses.

*p < 0.10.

**p < 0.05.

^***^ *p* < 0.01.

On average, medical students require €13,515/year in additional compensation to accept a job in their preferred foreign European country rather than in Italy, all else being equal. This is a considerable amount, as it corresponds to roughly 30% of the average starting annual net income of Italian physicians or to one third of the standard deviation of the distribution of average yearly net income of physicians across European countries reported by OECD (2019). Considering that moving abroad entails both monetary and non‐monetary costs, this result is not surprising.^9^

Figure 1 reports the distribution of the posterior individual level WTPs for working abroad, estimated using the method proposed by Revelt and Train (2000), and reveals clear heterogeneity in the estimated WTPs.

**FIGURE 1:**
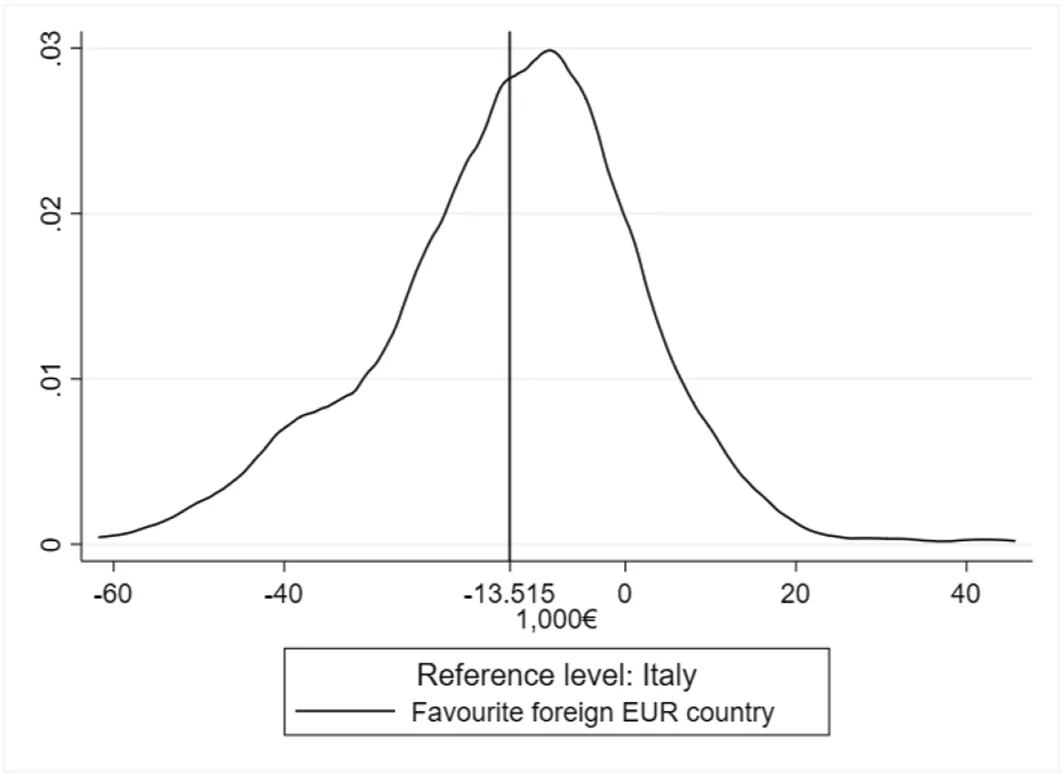
Density of the WTP distribution for jobs located in respondents' favorite European country. The vertical bar indicates the mean of the distribution.

The attribute non‐attendance analysis reported in Supporting Information S1: Online Appendix G further qualifies the interpretation of this heterogeneity. In that exercise, non‐attendance is modeled as a zero coefficient, and therefore a zero WTP, for the non‐attended attribute. The inferred probability of non‐attendance is highest for job location, suggesting that some respondents' choices can be rationalized as if country had no independent effect on utility, conditional on the other job attributes. We interpret this result as consistent with substantial heterogeneity in the salience of the Italy‐versus‐abroad distinction. This interpretation is supported by self‐reported attribute‐importance rankings collected after the choice tasks and reported in Supporting Information S1: Online Appendix Figure A3: country is ranked as the most important attribute by about 20% of respondents and among the two most important attributes by about one third of respondents, but also as the least important attribute by about 27%. Importantly, the average WTP for working abroad remains negative and close to the baseline estimate across the attribute non‐attendance specifications (see Supporting Information S1: Online Appendix G), suggesting that the main migration‐preference result is robust.

We next examine observable characteristics that predict this heterogeneity. The results, reported in Figure 2 are obtained from estimating Equation (4), and each row is for a different trait. Characteristics for which the mean WTP difference across groups is significant are marked with significance stars.^10^ Because these subgroup analyses were not separately powered to detect small differences across all individual characteristics, we interpret them as descriptive evidence of observed heterogeneity rather than as definitive tests of all possible predictors of migration intentions.

**FIGURE 2:**
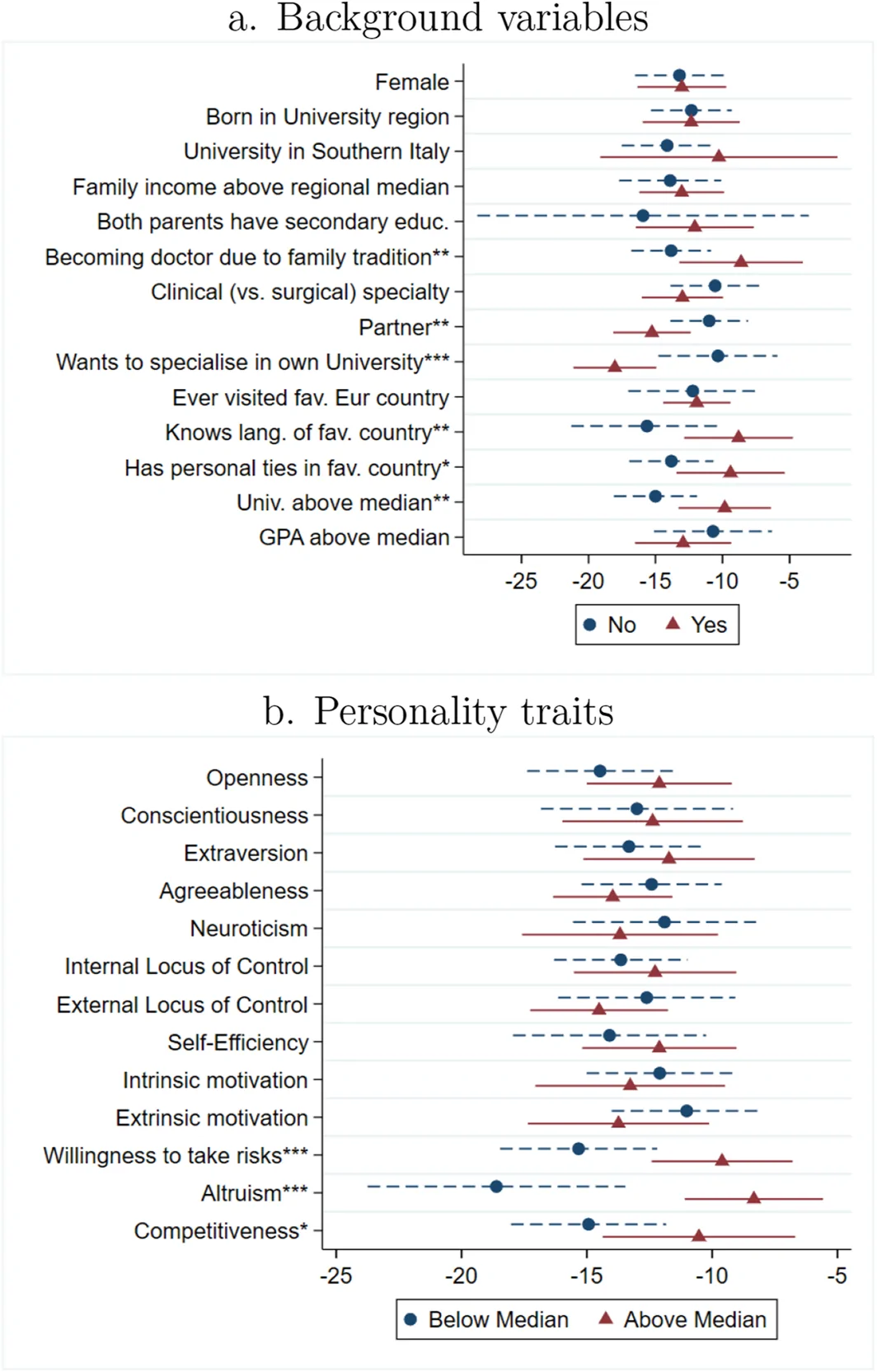
Mean of WTP distribution for jobs located in respondents' favorite foreign European country, by individual background variables and personality traits. (a) Background variables. (b) Personality traits.

Within our sample of highly‐educated medical students there is no evidence of gender differences in migration intentions, in line with findings in Docquier et al. (2009) and Docquier et al. (2012).

Similarly, we do not detect significant WTP differences by family income, parental education, early internal migration (attending medical schools outside of the region of birth), location of the attended University, or intended specialty choice (clinical vs. surgical).

Instead, students who want to pursue medicine to continue a family tradition display greater willingness to migrate. So long as valuable occupation‐specific knowledge is transmitted within the family, this finding suggests a potential negative selection of the domestic physician pool.

Individual‐level push and pull factors, as well as correlates of migration preferences, also matter. Students with a partner or planning to specialize at their current university are significantly less willing to move abroad, while those who know the language of their preferred country or have personal ties there are more willing.

Finally, an important matter concerns whether student ability predicts migration intentions (Bütikofer and Peri 2021; Nejad and Schurer 2022). Admission to Italian medical schools is based on a national entry test, with higher‐scoring students enrolling in universities with more selective thresholds. Several research institutes provide annual rankings of medical schools, the most widely used being the CENSIS index. We use attendance at a medical school above the sample‐median CENSIS score as a proxy for high ability.^11^ We find that students attending above‐median quality universities are significantly more willing to migrate, with potentially important implications for the skill composition of doctors remaining in Italy.^12^

By contrast, GPA within universities is not a good predictor of migration intentions, likely because ability sorting occurs at entry, leaving little within‐school variation.

Turning to personality traits, we find only limited effects of the Big Five. Medical students with higher openness and extraversion show somewhat greater willingness to migrate, but the differences are not significant. Similarly, self‐efficacy and locus of control display small, non‐significant associations with migration intentions.

The existing literature in economics typically reports stronger associations between personality and migration outcomes. Conscientiousness, openness to experiences, extraversion, and adaptability are found to be positively related to migration intentions and actual migration behavior (Fouarge et al. 2019; Bütikofer and Peri 2021; Nejad and Schurer 2022), and so is locus of control (Caliendo et al. 2019). The limited impacts of personality traits in our DCE compared with these studies may reflect differences in research design. As shown by Fouarge et al. (2019), personality plays a central role to explain migration to culturally distant locations, but not for destination countries perceived as close by subjects. In addition, we are investigating heterogeneous effects by personality within a sample of highly‐skilled and highly educated individuals, where there may be less variation in personality than in the general population.

Despite this limitation, some traits clearly play an important role. Consistent with Jaeger et al. (2010), we find that risk tolerance is positively associated with migration intentions. Altruism also plays a role: more altruistic students display a substantially higher willingness to migrate. Altruistic doctors are typically more inclined to forego personal benefits for patients, and Li (2018) show how altruistic preferences affect specialty choice and the self‐reported likelihood of practising in under‐served areas.^13^ Our results suggest that altruistic students may also be more likely to move abroad, possibly because they perceive greater opportunities to realise their professional and moral motivations. Competitiveness shows a similar pattern. Students with higher levels of competitiveness are more willing to migrate. This finding resonates with Hao et al. (2016), who show that migrants are more likely to enter competitive environments than non‐migrants. Buser et al. (2026) provide evidence that competitiveness is a strong and consistent predictor of labor market success in terms of both income and occupational quality. Our results suggest that migration may be one of the channels through which more competitive individuals pursue professional advancement.

Overall, migration seems more attractive to high‐ability medical students, those whose parents are doctors themselves, the more risk‐tolerant, altruistic, and competitive—raising concerns about the negative selection of the remaining physician workforce in terms of these skills and traits.

### 5.2 WTPs for Job Attributes and Their Heterogeneity by Job Location

Table 2 also reports the estimated WTPs for the other job attributes considered in the DCE. We find that medical students have a mean WTP of €6572 for having some development opportunities and a much higher WTP of €10,998 for having good development opportunities with respect to the baseline of poor development opportunity jobs. Job security is also important: compared to the reference level of a fixed‐term two‐year job contract with no renewal chance, a two‐year contract with a 50% renewal chance has a higher valuation amongst the students, with a mean WTP of €11,800 and €8878 for that of a permanent contract.^14^ As workers may quit a permanent job, the higher WTP for fixed‐term renewable jobs than for permanent jobs may seem puzzling. A possibility is that medical students may fear a stigma related to quitting a permanent job. When it comes to skill mismatch, we see that—with respect to other job attributes—students have a low willingness to pay for avoiding a job for which they are over‐skilled. This may partly depend on the fact that we are offering jobs as medical specialists, and students may perceive that there is limited potential for skill mismatch. In addition, we see that working conditions are highly valued, and students are willing to pay a high sum of €12,740 to have a job with adequate workload with little overtime work and night shifts as opposed to having a high workload with frequent overtime work and night shifts. Finally, the positive coefficient of 0.126 relating the proposed salary to the mean of the preference distribution suggests that the probability of choosing a job increases with higher salary.

To assess the relative importance of these job characteristics more formally, we test the equality of selected WTP estimates from the baseline model. Since WTP estimates are defined relative to the reference level of each attribute, these tests compare specific improvements in job characteristics rather than the intrinsic importance of attributes in the abstract. We focus on the three highest‐valued job improvements in Table 2: moving from poor to good working conditions, moving from a non‐renewable fixed‐term contract to a contract with a 50% renewal probability, and moving from limited to good professional development opportunities. Pairwise tests show that these three WTP estimates are not statistically different from one another at conventional levels; a joint test of equality also fails to reject the null hypothesis ($\chi^{2}\left(2\right)=2.70$, p=0.260). This suggests that students attach similarly high value to these three dimensions of job quality.

We then compare the WTP for exact skill‐job match and the compensation required to accept a job abroad with the average WTP for these three high‐valued improvements. The WTP for exact skill‐job match is significantly lower than this benchmark, with a difference of −€8135 per year (p<0.001). By contrast, the compensation required to accept a job abroad is not statistically different from the average WTP for the three highest‐valued improvements, with a difference of €1670 per year (p=0.233). These comparisons suggest that working conditions, job security with renewal prospects, and professional development opportunities are all important margins for improving the attractiveness of medical jobs. They also indicate that the disutility associated with working abroad is of the same order of magnitude as the most valued job‐quality improvements.

The magnitude of these WTP estimates is economically large. The average starting net income of Italian physicians in our setting is approximately €45,500 per year; hence, the WTPs for the most valued job improvements correspond to roughly one quarter to one third of annual starting income. These magnitudes are large, but not out of line with related DCE evidence. Direct numerical comparisons should be interpreted cautiously, because studies differ in populations, institutional settings, income measures, and attribute definitions. Nevertheless, the comparison is informative. Among practising physicians, previous studies find large valuations for workload and location‐related attributes. Scott (2001), studying UK general practitioners, reports monetary valuations of about £13,500 and £19,700 for changes in out‐of‐hours work commitments, consistent with the large value we find for moving from poor to good working conditions. Scott et al. (2013), studying experienced Australian general practitioners, estimate that moving to rural areas would require substantial financial incentives, ranging from about 37% of annual earnings for less remote rural towns to up to 130% of annual earnings for the least attractive rural job packages.

Evidence on students, trainees, and early‐career health workers points in a similar direction, while placing greater emphasis on career‐development opportunities. Sivey et al. (2012), studying Australian junior doctors, show that increasing GP earnings by AUD 50,000 or improving procedural or academic opportunities increases the predicted probability of choosing general practice by a similar amount, between 8 and 13% points. Cleland et al. (2016), studying UK foundation doctors and doctors in training, find that trainees would require compensation equivalent to 49.8% of average specialty earnings to move from good to poor working conditions, 38.4% to move from good to limited partner opportunities, and 30.8% to move from a desirable to a less desirable location. Holte et al. (2015), studying Norwegian final‐year medical students and interns, find that young doctors value control over working hours and professional development highly, and that income increases alone are insufficient to compensate for poor rural job attributes. Mandeville et al. (2016), studying junior doctors in Malawi, report very large valuations for specialty and training attributes, with some compensating differentials close to a doubling of basic salary. Kolstad (2011), studying clinical officer finalists in Tanzania, similarly shows that education and upgrading opportunities are among the strongest instruments for attracting early‐career health workers to rural jobs, with WTP estimates for early education opportunities exceeding the starting monthly wage.

Taken together, these studies suggest that our WTP estimates are economically large but plausible. They also help interpret how medical students value job amenities compared with more senior health professionals. Like practising physicians, students place high value on working conditions. Unlike more established physicians, however, students are at the beginning of their careers and therefore attach substantial value to attributes related to career formation, such as professional development opportunities and contracts with renewal prospects. Non et al. (2022), in a DCE on science and engineering graduates with job attributes similar to ours, estimate that avoiding frequent overtime and obtaining a permanent contract are worth about €328 and €254 per month, respectively, against monthly salaries ranging between €1800 and €2200. These comparisons suggest that our WTP estimates are economically substantial, yet remain within a plausible range.

Figure 3 plots the posterior distributions of the individual‐level WTPs of other job attributes. Like for job location, the evidence points to considerable unobserved heterogeneity. For instance, it is remarkable to see that individuals' WTP for a permanent job is markedly heterogeneous, and spans a large range of positive and negative values.

**FIGURE 3:**
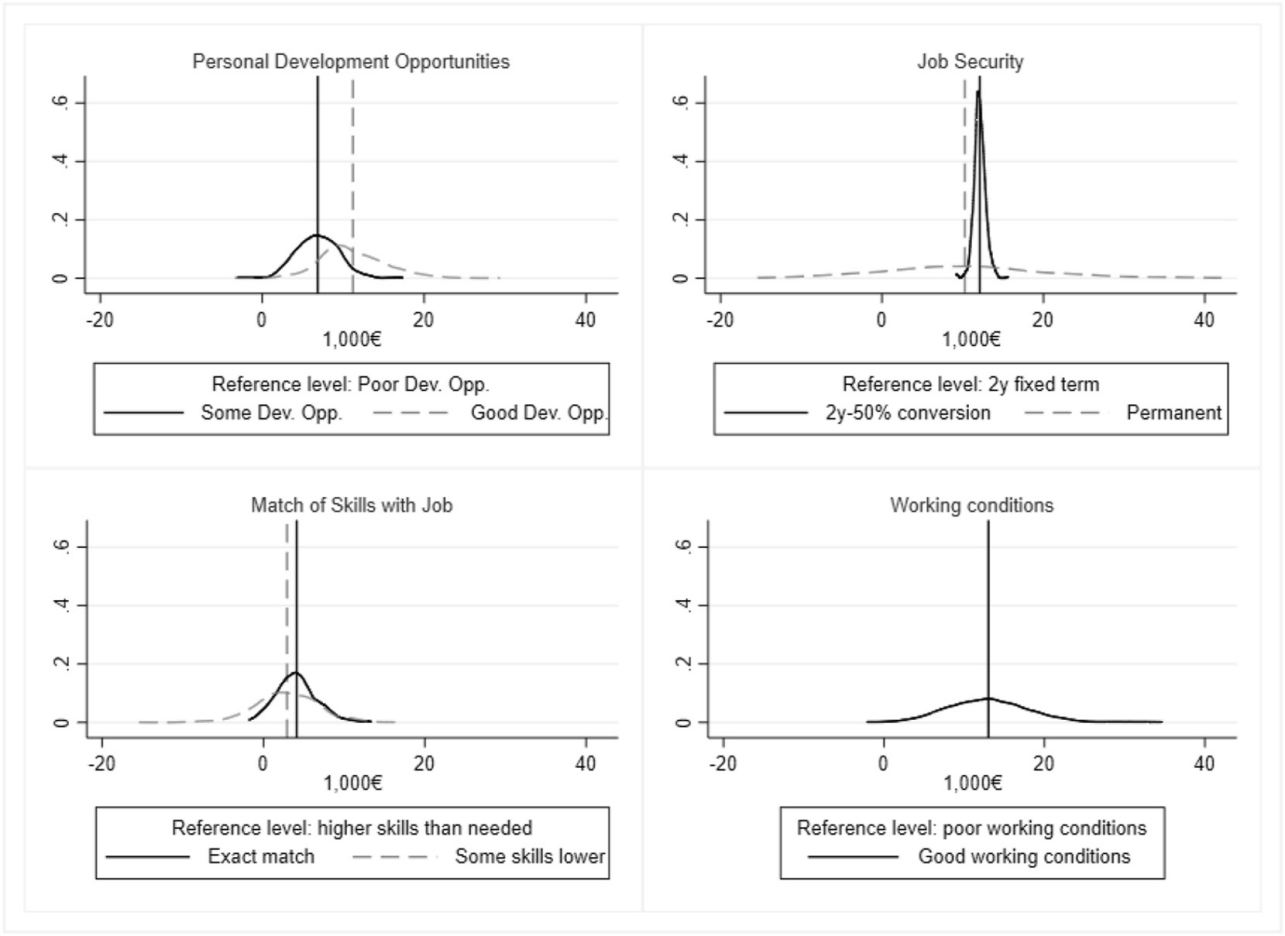
Density of the WTP distribution for non‐monetary and non‐geographical job attributes. Vertical bars indicate the mean of each distribution.

We next investigate whether this unobserved variability depends on the heterogeneity of medical students' preferences for the attributes of jobs located in their home country or in their favorite foreign European country. In Table 3, we report the WTP estimates of job characteristics for jobs located home and abroad obtained from Equation (5), and test whether the differences are statistically significant.

**TABLE 3:** Mean WTP estimates for non‐monetary,non‐geographical job attributes across jobs located in Italy and in respondents' favorite foreign European country (abroad).

Variable	Home	Abroad	Difference
Professional development opportunities WTP—reference: poor dev. opp.			
Some dev. opp.	13,563***	−2031	−15,594***
	(2098)	(1977)	(3320)
Good dev. opp.	11,269***	7137***	−4132***
	(2090)	(1546)	(2434)
Job security WTP—reference: fix‐term,non‐renewable job			
Fixed‐term job, 50% renew prob.	3866***	20,978***	17,111***
	(1441)	(1275)	(1934)
Permanent job	13,979***	1888	−12,091***
	(1834)	(1877)	(2809)
Working conditions WTP—reference level: poor			
Good working conditions	13,243***	12,086***	−1157
	(1243)	(1391)	(1999)
Match of skills with job content WTP—reference: higher than needed			
Exactly matched	8219***	−2165*	−10,384***
	(1545)	(1279)	(2118)
Lower than needed	9085***	−4209***	−13,294***
	(1453)	(1542)	(2351)
Choices	8432		
Subjects	527		

*Note:* This table reports estimates obtained from Equation (5). $ASC_{1}$ and $ASC_{2}$ are alternative‐specific constants for the first and second job offers, capturing position effects (i.e., systematic tendencies to prefer the left or right alternative irrespective of its attributes). Observations are weighted to match the population level statistics of the share of females, age and share of students by macro‐regions. Robust standard errors in parentheses.

^*^ *p* < 0.10.

**p < 0.05.

^***^ *p* < 0.01.

Except for good working conditions, we find significant WTP differences between home and abroad in every job attribute level. We see that medical students have a very high WTP for a permanent job contract at home, but would prefer a temporary job contract with renewal possibility in their favorite foreign European country. As in the pooled case, the non‐linear effect of job security may be related to the fear of stigma effects relating to quitting from a permanent job abroad. We also learn that, consistently, professional development opportunities are highly valued by them for jobs located at home, but not as much for jobs located abroad. Finally, we see that they do not mind being under‐skilled in jobs located in their home country, but are really averse to this in foreign countries. This pattern may be consistent with respondents perceiving up‐skilling in a foreign country—where they may also face other difficulties, concerning linguistic or cultural integration—to be harder or more costly, therefore preferring to not take jobs where their skills are lower than needed.

Overall, the results show that medical students in our sample are more prone to permanently settling down in their home country than in their favorite foreign European country. They are instead willing to go abroad for a short period of time to take up jobs that do not seem too challenging for them and explore the life and work conditions there, with the option of settling there for the long‐term.

### 5.3 Policy Simulations: Predicted Choice Probabilities Under Alternative Job Offers

Table 4 reports selected policy simulations based on the baseline mixed logit model. The baseline scenario assumes that both jobs offer €40,000, limited professional development opportunities, a two‐year fixed‐term non‐renewable contract, high workload with frequent overtime work and night shifts, and exact skill match. In this scenario, the Italian and foreign jobs have identical observed characteristics except for location. Subsequent scenarios change one attribute of the Italian job while holding the foreign job at the baseline.

**TABLE 4:** Policy simulations: conditional predicted choice probabilities under selected job‐offer scenarios.

Scenario	Italian job	Foreign job	Conditional predicted choice probability (%)	SE (pp)	Change from baseline (pp)
Italian job becomes more attractive			P(choose Italian job)		
Baseline: Identical low‐amenity jobs except for location	€40,000; limited development; 2‐year fixed‐term non‐renewable contract; high workload; exact skill match	Same attributes abroad	69.9	1.8	—
Good professional development in Italy	Good development opportunities; otherwise baseline	Baseline foreign job	80.9	1.6	11.0
Permanent position in Italy	Permanent position; otherwise baseline	Baseline foreign job	77.1	1.5	7.1
Adequate workload in Italy	Adequate workload with little overtime and night shifts; otherwise baseline	Baseline foreign job	82.1	1.4	12.2
Italian salary €50,000	€50,000; otherwise baseline	Baseline foreign job	81.0	1.5	11.1
Italian salary €60,000	€60,000; otherwise baseline	Baseline foreign job	88.4	1.0	18.4
Italian salary €75,000	€75,000; otherwise baseline	Baseline foreign job	94.2	0.5	24.3

*Note:* The table reports conditional predicted choice probabilities from the baseline mixed logit model. The baseline scenario assumes that both jobs offer €40,000, limited professional development opportunities, a two‐year fixed‐term non‐renewable contract, high workload with frequent overtime work and night shifts, and exact skill match; the two jobs differ only by location. Each subsequent scenario changes one attribute of the Italian job, while holding the foreign job at the baseline. Predicted probabilities are reported as percentages. Standard errors are reported in percentage points. Changes from baseline are absolute differences in percentage points. These probabilities are conditional on choosing between the two job offers and should not be interpreted as direct forecasts of aggregate migration flows or actual retention rates.

Abbreviation: pp, percentage points.

Under the baseline comparison, the predicted probability of choosing the Italian job is 69.9%, while the predicted probability of choosing the foreign job is 30.1%. This confirms the sizable average preference for remaining in Italy documented in the WTP estimates.

Table 4 shows how improvements in the Italian job offer affect the conditional probability of choosing the Italian job. Good professional development opportunities increase the predicted probability of choosing Italy from 69.9% to 80.9%. Improving job security by offering a permanent position increases the predicted probability to 77.1%, while improving working conditions from high workload to adequate workload increases it to 82.1%. Salary increases have particularly large effects: increasing the Italian salary to €50,000, €60,000 and €75,000 raises the predicted probability of choosing the Italian job to 81.0%, 88.4% and 94.2%, respectively.

These simulations complement the WTP and location‐interaction analyses by showing how average preferences translate into predicted choices under stylized job‐offer scenarios. The policy simulations therefore provide a practical summary of how improvements in Italian job offers may affect the relative attractiveness of staying in Italy within the experimental choice setting.

### 5.4 Heterogeneity in the Valuation of Selected Job Amenities

We next examine whether the valuation of selected job amenities differs across groups of students. We do not replicate the full heterogeneity analysis for every attribute level, as this would generate a large number of comparisons and would be difficult to interpret. Instead, we use the baseline WTP distributions in Figure 3 to guide the choice of attributes. We focus on attribute levels for which there is substantial dispersion in WTPs or for which the sign and magnitude of valuations are less uniform across respondents: a permanent job, good professional development opportunities, and a job in which some skills are lower than required.

These attributes are particularly informative because they capture dimensions on which students may plausibly disagree. A permanent contract may be valued as job security by some students, but as reduced flexibility by others. A job requiring further skill development may be perceived either as a challenge and learning opportunity or as a source of risk. Professional development opportunities may also matter differently depending on students' career orientation and motivations. By contrast, the WTP distributions for a contract with a 50% renewal probability, exact skill‐job match, and good working conditions are concentrated on positive values, suggesting broad agreement that these are desirable job improvements. We therefore discuss these attributes primarily through the baseline WTP estimates and the equality tests reported above, while focusing the heterogeneity analysis on attributes for which cross‐student differences are more informative.

Figures 4, 5 and 6 report heterogeneous mean WTP estimates for the three selected attributes, distinguishing between background characteristics and personality traits as in Figure 2. The results show that the valuation of these job amenities is not confined to a narrow subgroup of students. At the same time, they reveal meaningful heterogeneity.

**FIGURE 4:**
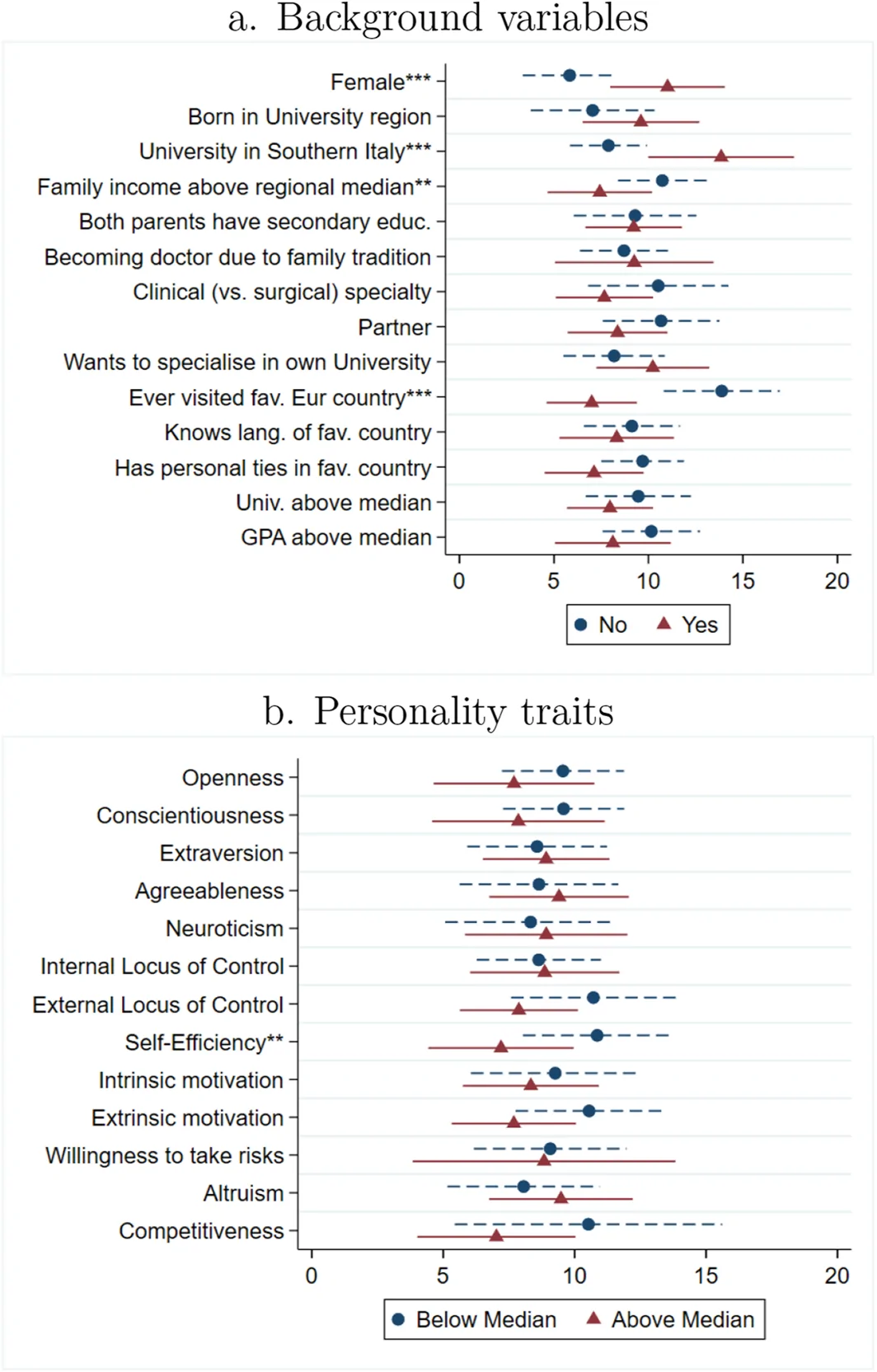
Heterogeneous mean WTP for a permanent job, by individual background variables and personality traits. (a) Background variables. (b) Personality traits.

**FIGURE 5:**
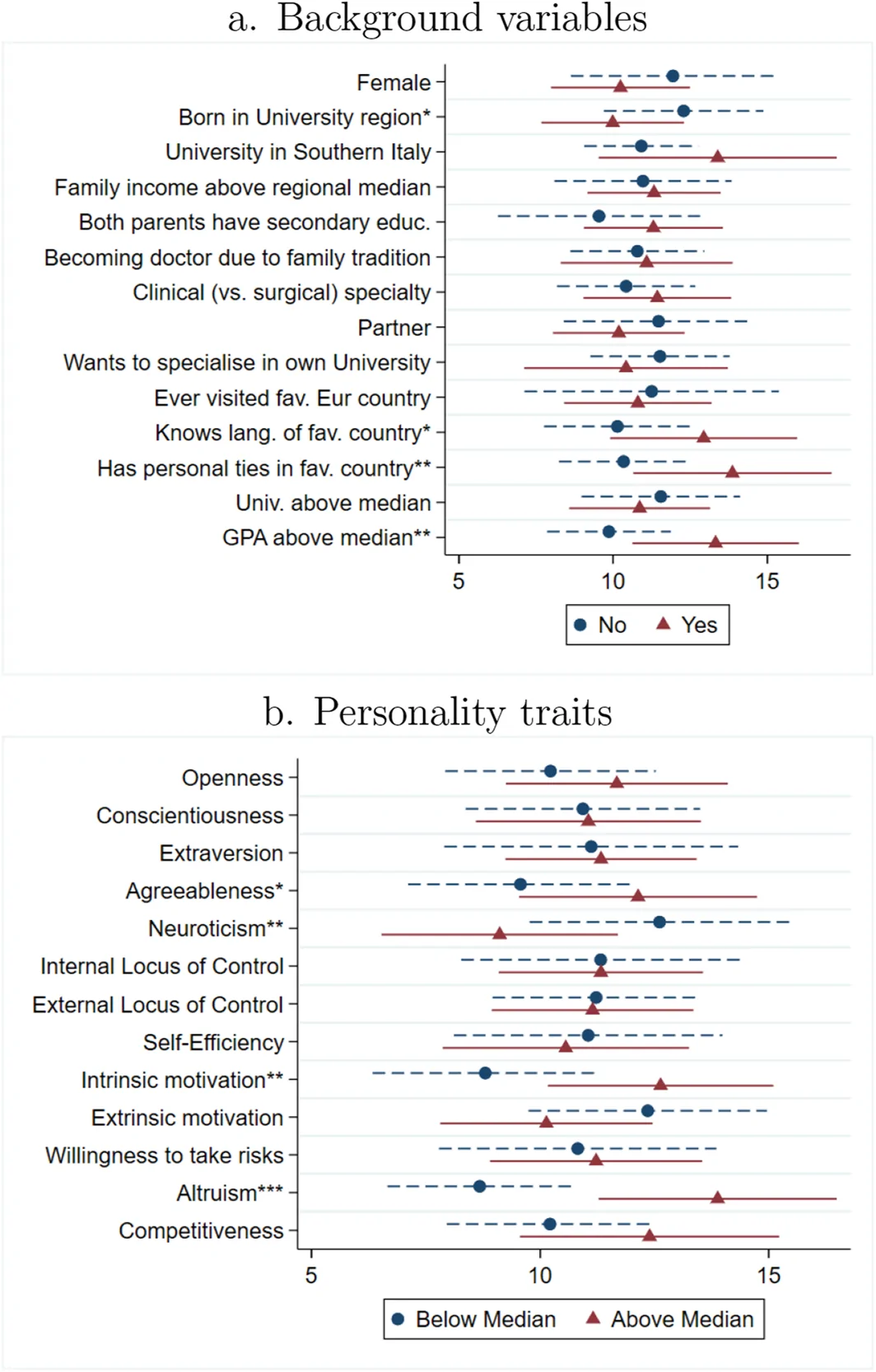
Heterogeneous mean WTP for good professional development opportunities, by individual background variables and personality traits. (a) Background variables. (b) Personality traits.

**FIGURE 6:**
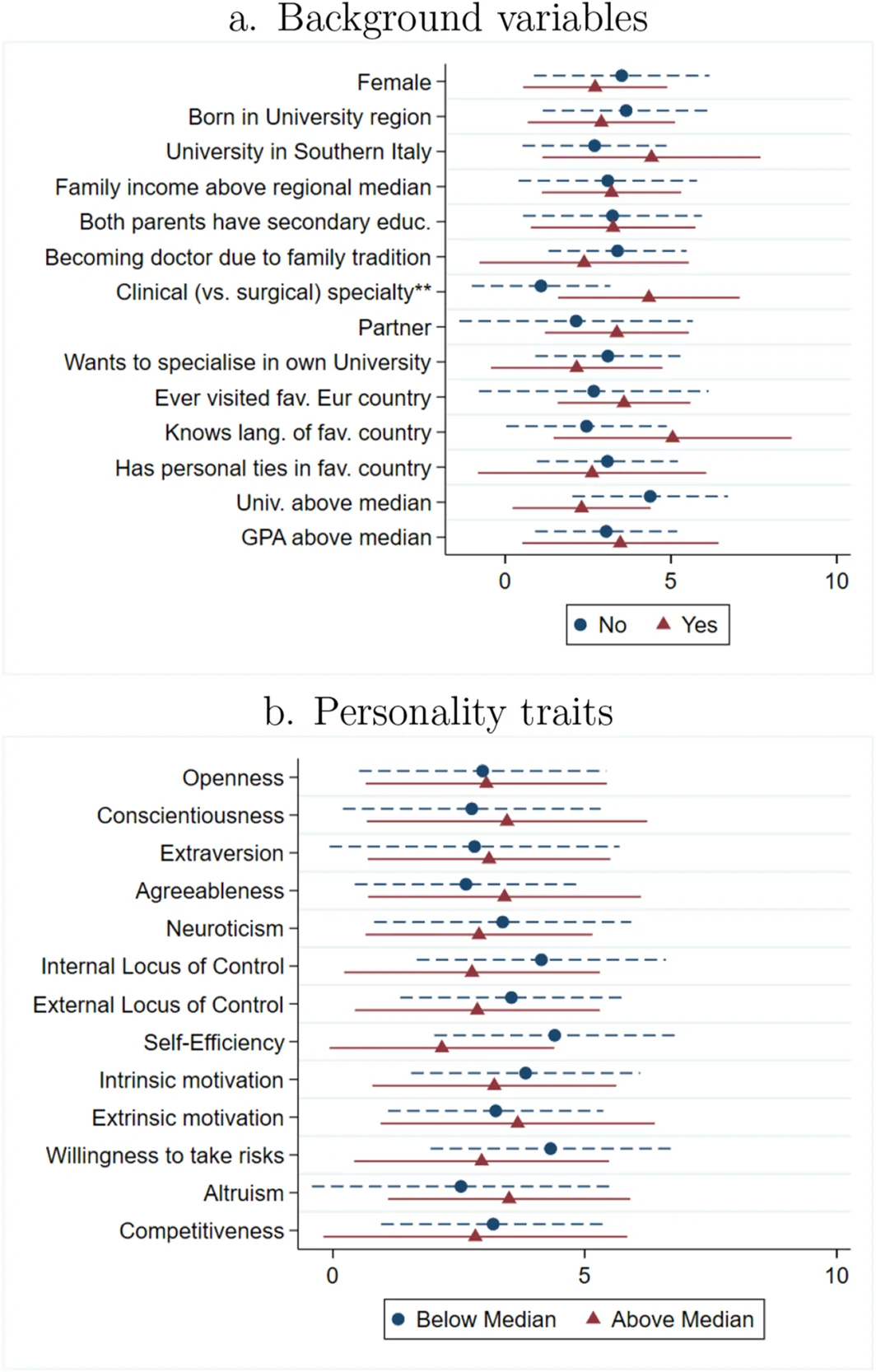
Heterogeneous mean WTP for jobs in which skills are lower than required, by individual background variables and personality traits. (a) Background variables. (b) Personality traits.

For permanent jobs, the WTP is positive for most groups but differs substantially across student characteristics. Contractual stability appears more valuable for women and for students enrolled in universities in Southern Italy, while it is less valuable for students with characteristics associated with greater mobility or stronger outside options, such as higher family income, attendance at higher‐quality universities, or higher openness. This suggests that job security is an important retention margin, but not equally salient for all students.

For good professional development opportunities, WTPs are positive and sizable across most groups. The estimates tend to be higher among students with stronger intrinsic or prosocial motivations, such as those with higher intrinsic motivation and altruism. This is consistent with the interpretation that training and research opportunities are particularly attractive to students who attach value to the professional and developmental content of medical work.

Finally, the WTP for jobs in which some skills are lower than required is smaller and more heterogeneous. The confidence intervals often span both positive and negative values, suggesting less agreement across respondents on how to value this attribute. This pattern is consistent with the ambiguous nature of the attribute: some students may perceive such jobs as opportunities for learning and professional challenge, while others may perceive them as risky or costly positions.

Overall, this analysis complements the heterogeneity results for job location. Students differ not only in their willingness to move abroad, but also in the job attributes they value most. This reinforces the policy relevance of considering multiple margins of job quality when designing retention policies.

### 5.5 Robustness Checks

In Supporting Information S1: Online Appendix F we report the results of several sensitivity tests. First, Supporting Information S1: Online Appendix Tables A5 and A6 report results when we exclude respondents whose favorite European country is the UK—no longer an EU member since 2020. Second, Supporting Information S1: Online Appendix Tables A7 and A8 report the estimates obtained without the use of sampling weights. Third, Supporting Information S1: Online Appendix Tables A9 and A10 show the estimates using a conditional logit model—that overlooks the heterogeneity of individual preferences—instead of the mixed logit one. As can be seen, our main qualitative conclusions remain unchanged.^15^ Supporting Information S1: Online Appendix Tables A11, A12, and A13 additionally report baseline WTP estimates for the subsamples of respondents who rated the tasks as very clear, not difficult, and both very clear and not difficult, respectively; the estimates are broadly comparable to the full‐sample baseline. Finally, given that our choice task requires students to trade off among several attributes, in Supporting Information S1: Online Appendix G we investigate the potential role played by attribute non‐attendance (see also Arora et al. 2022) using the approach based on latent class models championed by Scarpa et al. (2009), and the results confirm our main ones. Supporting Information S1: Online Appendix Figure A4 additionally reports how our main mean WTP estimates change when we drop one university at a time. The baseline estimates are reassuringly stable across these leave‐one‐university‐out specifications.

## 6 Conclusion

This paper used a Discrete Choice Experiment on medical students from 12 Italian universities to analyze how individual traits and preferences for job attributes shape their migration intentions.

We find that, on average, medical students require additional compensation of about €13,500 per year to accept a job abroad rather than in Italy, all else being equal, an amount equal to roughly 30% of Italian physicians' starting net income. Beyond this aggregate valuation, background characteristics (such as attending a high‐quality medical school or coming from a medical family) and personality traits (such as altruism, risk tolerance, and competitiveness) predict which students need less compensation to migrate. In addition, job preferences differ systematically by location: students strongly value permanent contracts and professional development opportunities at home, while abroad they accept temporary contracts with renewal options but are averse to being under‐skilled.

These findings have two main implications. First, medical emigration disproportionately concerns the most well‐trained students, with potentially severe consequences for sending countries. Second, although students display a strong willingness to sacrifice wages to stay in Italy, the persistent outflows indicate that domestic jobs do not provide the stability and career prospects they seek, or that the monetary compensation for giving up on those job attributes is too low. This gap calls for policy interventions to redesign medical jobs. Our results suggest that students attach economically large value to several dimensions of job quality, including working conditions, job security with renewal prospects, and professional development opportunities. The heterogeneity analysis further shows that these margins are not equally salient for all students: contractual stability, professional development, and jobs requiring further skill development appeal to different groups. Since these margins may differ substantially in their fiscal and organisational costs, policymakers can view them as alternative or complementary retention tools and prioritize the mix of interventions that is most feasible and cost‐effective in their institutional context.

A limitation of our study concerns the treatment of personality traits in the heterogeneity analysis. We measure traits using survey indicators and constructed indices, and then include these indices as observed characteristics when examining heterogeneity in WTP for working abroad. Although the items used to construct these indices are based on established survey measures, they may still contain measurement error. As a result, the estimated associations between personality traits and migration preferences may be attenuated, and the limited statistical evidence for some traits should not necessarily be interpreted as evidence that these traits are irrelevant. A more structural approach would be to estimate a hybrid choice model, in which personality traits are treated as latent constructs measured through multiple indicators and linked jointly to the choice model. Such an approach could account more explicitly for measurement error in the personality indicators and may provide a richer understanding of how latent psychological characteristics shape migration preferences. We view this as a promising avenue for future research.

A further limitation concerns possible ordering effects in the DCE. The order of the 16 experimental choice tasks was fixed within each questionnaire version and was not randomised between respondents. Randomizing choice‐task order can help reduce potential choice‐set ordering effects, including learning or fatigue effects across the sequence of tasks (Boxebeld 2024). We therefore cannot fully rule out the possibility that the sequence in which choice tasks were presented influenced respondents' choices.

Needless to say, Italy is only one case, but it is a particularly important one: a high‐income country where medical brain drain is already undermining the sustainability of the healthcare system. Addressing these gaps is crucial not only for Italy but also for other countries facing shortages of healthcare professionals.

## Funding

Marco Bertoni and Debdeep Chattopadhyay acknowledge funding from a Cassa di Risparmio di Padova e Rovigo (CARIPARO) Starting Grant. Yuanyuan Gu acknowledges funding from a Marie Sklodowska‐Curie Individual Fellowship provided by the European Commission.

## Ethics Statement

The Human Research Ethics committee at Macquarie University granted IRB approval to the project on 10 March 2020. Reference No: 52020627314481, Project ID: 6273.

## Conflicts of Interest

The authors declare no conflicts of interest.

## Supporting information


Supporting Information S1

## Data Availability

The data that support the findings of this study are available from the corresponding author upon reasonable request.

## Endnotes

^1^
From this perspective, our investigation is related to the analyses of Batista and Mckenzie (2023)—who use lab experiments on samples in Lisbon and Nairobi to test how potential migrants trade‐off different attributes of potential migration destinations (including migration costs, returns to skills, unemployment risk and social benefits)—and of Özer and de Grip (2025)—who assess the differences in the valuation of several job characteristics of local jobs between job seekers from different countries. Unlike their analyses, however, we focus on the heterogeneous evaluation from the same individuals of attributes of jobs located in different countries.

^2^
Doiron and Yoo (2017) provide longitudinal evidence on the stability of non‐monetary job attribute valuations among nursing students as they transition from education to employment across survey waves.

^3^
Almalaurea is a consortium of Italian universities that collects systematic data on graduate employment outcomes.

^4^
For instance, working as a doctor in the US requires to pass a formal exam, and we did not want students to make assumptions on this. We eventually discarded from the final sample 2 students who listed a non‐European country as their favourite country. Despite Brexit, we retained students who chose the UK, but we will show that our results hold irrespective of their inclusion.

^5^
Put differently, by asking students to name their favourite foreign country, we placed them in a situation where, conditional on intending to migrate, they had already maximized expected utility with respect to non‐work related expected benefits and costs across European countries.

^6^
A related concern is whether respondents were aware of the average level of the attributes in the countries involved, and thus might have considered some scenarios implausible. While attribute levels may differ from national averages, none of the combinations we proposed was unrealistic within the European context.

^7^
The use of demographic variables (age, gender) and geographic area to estimate sample weights is common in many general population surveys, including the Survey of Health and Retirement in Europe—SHARE, the Bank of Italy's Survey of Household Income and Wealth, the UK's Understanding Society.

^8^
We set the number of draws to 5000 after verifying the stability of the estimated WTPs to a different number of draws ranging from 1000 to 5000.

^9^
Importantly, a consequence of the PPP assumption that we asked respondents to maintain is that the monetary costs of moving do not vary depending on the chosen destination country.

^10^
The corresponding SDs are reported in Supporting Information S1: Online Appendix Figure A2.

^11^
Because the entry test is taken at the end of high school, the score reflects both innate ability and the effects of prior investment in education.

^12^
This result could also reflect university effects: top schools may shape students' migration intentions. While we cannot distinguish between selection and treatment, both imply negative selection of the domestic physician pool.

^13^
See Galizzi et al. (2015) for a review of the economic literature on altruism in the medical profession. Attema et al. (2023) discuss how patient‐regarding altruism is shaped by medical education.

^14^
The *Controesodo* policy, offering tax breaks to college‐graduate emigrants returning in Italy after 2 years abroad, may have incentivised temporary migration. However, students' higher WTP for 2‐year renewable over fixed‐term jobs abroad indicates their preferences are not solely driven by policy eligibility.

^15^
We have also estimated the baseline mixed logit model in the preference space. The implied WTPs distributions, obtained using simulations, are comparable to those from WTP space‐estimation, and are not reported.
