# Supplementary material for: Medical Brain Drain: The Role of Job Attributes and Individual Traits
Source: Health Econ. 2026 Aug 24;35(12):1880–98. doi: 10.1002/hec.70148 (PMC13724284; doi:10.1002/hec.70148)
Supplement: Supplementary file 1 [file HEC-35-1880-s001.pdf]

Medical Brain Drain: The Role of Job Attributes and Individual Traits

Marco Bertoni, Debdeep Chattopadhyay, Yuanyuan Gu

SUPPLEMENTARY MATERIAL - FOR ONLINE PUBLICATION ONLY

A Online Appendix A. Additional figures and tables

Figure A1: Choice task introduction and example

We now ask you to imagine that you have just completed your preferred specialization course and are looking for a job as a hospital doctor in your field of specialization. The next section will present a series of hypothetical scenarios. We will ask you to choose from some hypothetical job offers. Each scenario will contain two jobs, called "Offer A" and "Offer B". You will be asked to choose whether you prefer Offer A, Offer B, or neither. Each offer will be described using six characteristics, which we present below. Please read this information before proceeding.

1. Professional development opportunities. It refers to the opportunities for further research and training. Possible levels: Limited, Some, Good .

2. Annual net income. For open-ended contracts, the reported income is for the first two years, then assumes an increasing profile of about 5% every 2 years. If the work refers to a foreign country where the cost of living is higher (lower) than in Italy, assume to earn proportionally more (less) in order to maintain your purchasing power equal to what you would have in Italy with that sum. Possible levels: €20, 000, €30, 000, €40, 000, €50, 000, €60, 000, €75, 000.

3. Job security. It refers to contract duration. Possible levels: 2-year fixed-term contract non-renewable; 2-year fixed-term contract with 50% probability of a permanent position afterwards; Permanent position.

4. Working conditions. Possible levels: High workload, with overtime, availability and very frequent night shifts; Adequate workload, with overtime, availability and infrequent night shifts.

5. Match of skills with job content. Possible levels: Some of your skills are lower than those required by this job and need to be developed; Your skills are in line with those required by this job; Your skills are higher than those required by this job.

6. Country. Possible levels: Italy; Your favorite European foreign country.

Imagine that you have just completed your preferred specialization course and are faced with the following job offers for hospital doctor positions in your specialization. Which would you choose: A, B, or neither of the two?

	Job A	Job B	Opt-Out
Professional development Opportunity	Good opportunities for further research and training	Limited opportunities for further research and training	
Income (PPP adjusted)	€40,000	€40,000	
Job security	Permanent position	2-year temporary contract with 50% chance of a permanent position afterward	
Working Conditions	High workload with frequent overtime work and night shifts	Adequate workload with little overtime work and night shifts	
Match of skills with job content	Your skills are exactly matched to what is required by the job	Some of your skills are lower than required by the job and need further development	
Country	Your favourite foreign European country	Italy	
Which would you choose?	○	○	○

Figure A2: SD of WTP distribution for jobs located in respondents' favourite foreign European country, by individual background variables and personality traits.

a. Background variables

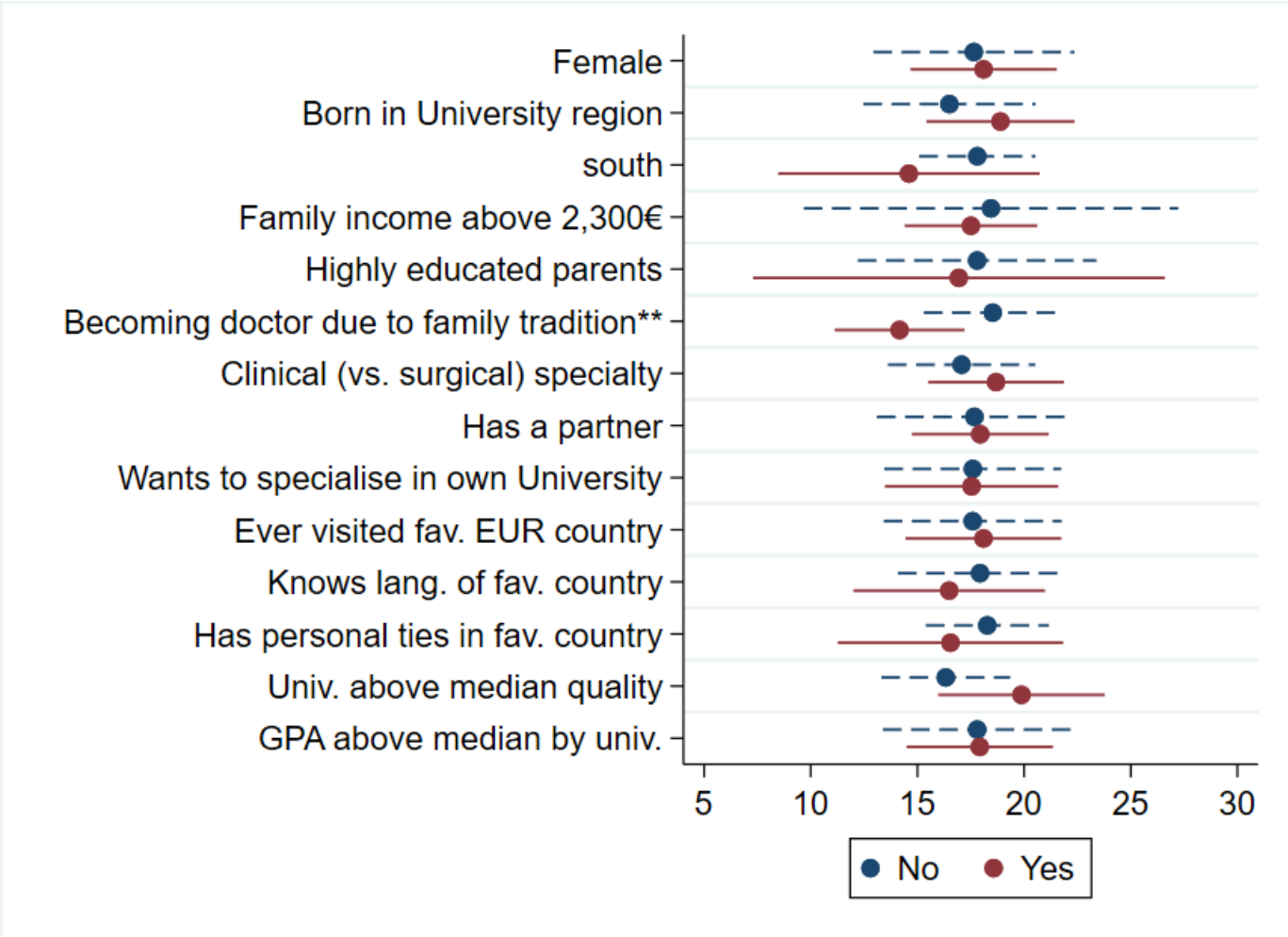

b. Personality traits

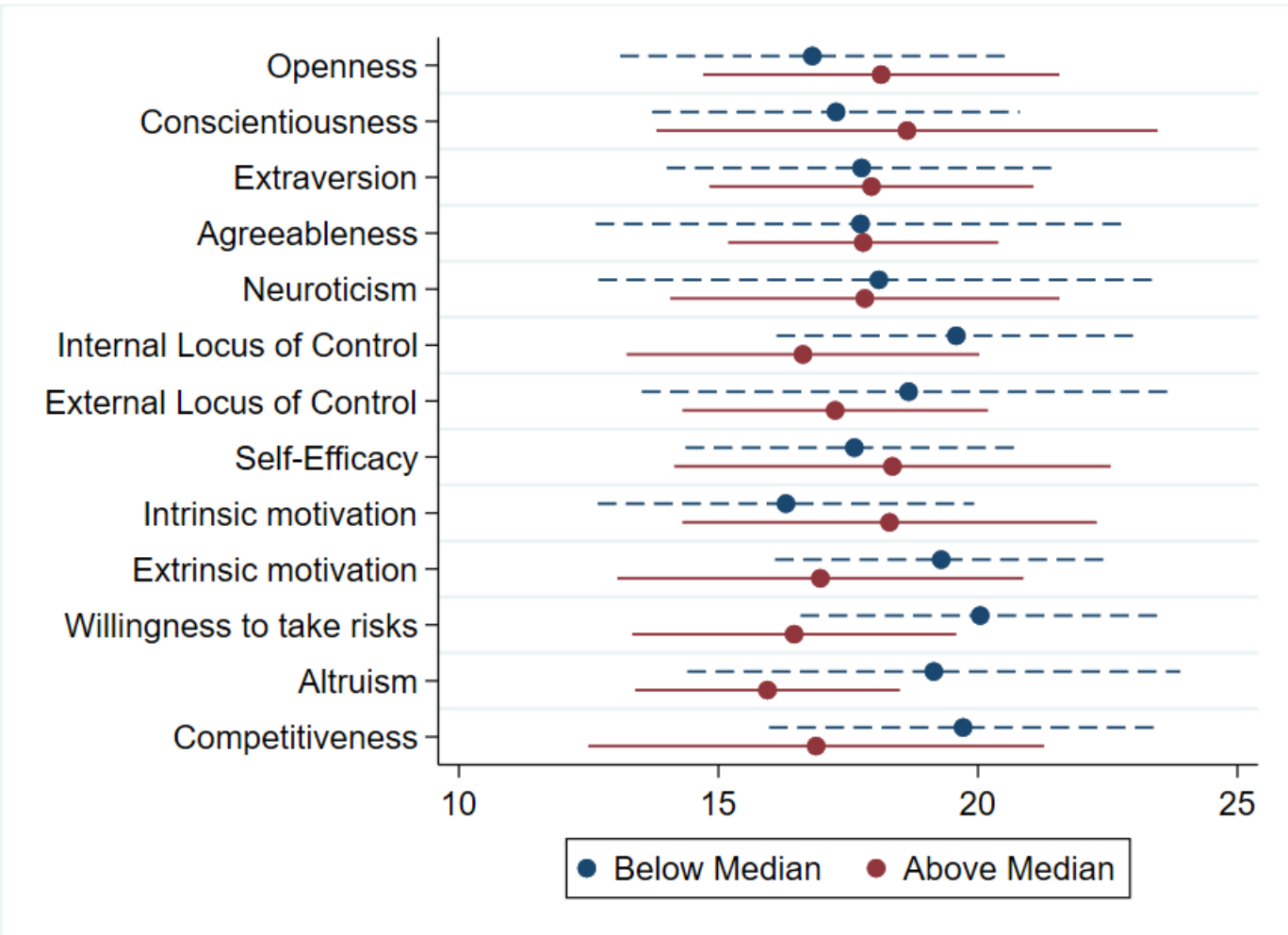

Table A1: Distribution of favourite foreign European countries in the final sample

Country	Sample share
United Kingdom	21.25
Spain	18.98
Switzerland	11.76
France	10.63
Germany	10.06
Netherlands	6.83
Portugal	3.23
Denmark	2.85
Ireland	2.66
Norway	2.66
Austria	2.47
Sweden	1.9
Belgium	1.52
Finland	1.33
Greece	0.57
Poland	0.38
Croatia	0.19
Cyprus	0.19
Luxembourg	0.19
Malta	0.19
Slovenia	0.19
Observations	527

Table A2: Distribution of respondents in the final sample by region and university

University	Sample size	Sample share
North-east		
Bologna	7	1.3
Ferrara	80	15.2
Padova	88	16.7
Udine	37	7.0
North-west		
Genova	37	7.0
Milano Statale	55	10.4
Centre		
Pisa	92	17.5
Roma "Campus biomedico" (private)	17	3.2
Roma "Sapienza"	11	2.1
South		
Bari	50	9.5
Catanzaro	5	1.0
Molise	48	9.1
Total	527	100.0

Table A3: Sample representativeness

Variable	Population mean	Sample mean	Reweighted sample mean
Female	0.524	0.588	0.525
Age	23.90	24.73	23.91
Student shares by region			
North-East	0.284	0.402	0.284
North-West	0.172	0.175	0.171
Centre	0.379	0.228	0.379
South	0.165	0.195	0.165
Observations	6,599	527	527

Table A4: Descriptive statistics

Variable	Sample mean	Reweighted sample mean
Female	0.588	0.525
Born in the university's region	0.571	0.606
Parents have a secondary education degree or higher	0.705	0.726
Family income above €2,300	0.590	0.619
Has a partner	0.619	0.602
GPA above median by university	0.410	0.444
Wants to specialise in own university	0.353	0.371
Clinical (vs.) surgical specialty	0.624	0.631
Ever visited fav. EUR country	0.729	0.744
Knows lang. of fav. country	0.324	0.324
Has personal ties in fav. country	0.264	0.259
Observations	527	

B Online Appendix B. Income levels definition

We decided the income levels based on data from the OECD (2019) on the average specialists' net income levels in European countries. After adjusting for PPP between Italy and the other countries, the observed average salaries vary from €31,600/year to €115,400/year, and the average Italian doctor's salary is €45,500/year. The key limitation of the OECD data is that they refer to the whole population of MDs, and therefore they do not represent starting salaries of medical school graduates immediately after finishing their specialisations. Anelli (2019) used Social Security (INPS) data for Italy to trace the earnings profiles of medical doctors. According to his data, the ratio of the earnings of doctors right after the end of specialisation and doctors at the middle of their career is roughly equal to two-thirds. In the absence of better data regarding the starting salaries of specialists in the different European countries, we use this principle to deflate the average salaries reported in the OECD data and compute reasonable starting salaries offered to newly specialised doctors. Under these assumptions, we use a lower bound for net entry yearly salary equal to €20,000 and the upper bound of €75,000. These income levels are all PPP adjusted using Italy as a benchmark. We also assumed that these PPP adjusted net income levels could appear across all countries considered in the study even though at the time of our survey the top (or low) end income levels may be more common in some countries than the others. Nevertheless, in Italy, the €75,000 income level is uncommon but nothing unheard of. For example, Anelli (2019) reports that among newly-specialised medical graduates there is a positive probability of being even in the top 1% of the income distribution in Italy. The relatively large income range also provides sufficient room for the elicitation of the underlying valuation of desirable jobs which may be not often seen at the time. This strategy is commonly used in the DCE studies for the purpose of product or policy design. In the meantime, to obtain a design with good properties such as efficiency and balance, we should refrain from imposing constraints to the experimental design unless certain combinations are *absolutely* implausible, which were not identified in our case.

C Online Appendix C. Survey instruments

This Online Appendix describes the survey instrument used to measure personality traits of respondents. Unless otherwise stated, answers to each item had to be reported on a 5-point ordinal Likert scale, with 1 meaning "strongly disagree" and 5 meaning "strongly agree". For each trait, we obtained a single index measure by standardizing the answers to each item in the final sample and then summing across items. Trait measures obtained with this simple method were highly correlated to the ones we estimated with polychoric principal component analysis (PPCA), but we preferred the former method because of convergence problems for the PPCA in some cases. We now turn to describe the survey instruments in detail.

– *Self-Efficacy*: We use three questions from Chen et al. (2001)

  1. I will be able to achieve most of my goals that I have set for myself
  2. Compared to other people, I can do most tasks very well
  3. I try to avoid competition with others (reversed)

  The original scale consists of 14 items. Non et al. (2022) exploit data collected on the full scale from university students (N=240) in a laboratory experiment and select three items that jointly have the highest correlation with the full scale (0.94 for the three mentioned items). In order to reduce survey length, we follow their selection.

– *Competitiveness*: We again follow Non et al. (2022) and use the following three (out of fourteen) items from the Revised Competitiveness Index:

  1. I try to avoid competition with others (reversed)
  2. I don't like competing against other people (reversed)
  3. I like competition

– *Locus of control*: We use a six-item scale as described in Lumpkin (1985)

  1. When I make plans, I'm almost sure that I'm going to make them work
  2. Getting people to do the right thing is a matter of skill, luck has nothing to do with it

3. What happens to me depends only on my choices
4. Many of the sad things in people's lives depend in part on bad luck
5. Getting a good job mostly depends on being in the right place at the right time
6. Many times, I feel that I have little influence on the things that happen to me

– *Big-5 personality traits*: We use an 11-item scale as used in the Survey of Health, Ageing and Retirement in Europe (SHARE). The following items are included in the scale.
  I see myself as a person...

  1. ...who is reserved
  2. ...who usually trusts
  3. ...who usually tends to be lazy
  4. ...relaxed, who handles stress well
  5. ...who has few artistic interests
  6. ...extroverted, sociable
  7. ...who tends to be laughed at by others
  8. ...who works accurately
  9. ...who gets nervous easily
  10. ...who has an active imagination
  11. ...caring and kind to almost everyone

  Items 5 and 10 relate to Openness; items 3 an 8 to Conscientiousness; items 1 and 6 to Extraversion; items 2, 7 and 11 to Agreeableness; items 4 and 9 to Neuroticism.

– *Intrinsic and extrinsic motivation*: We framed the following 7 questions after a detailed literature review on intrinsic and extrinsic motivation - see Ryan and Deci (2000); Kingston et al. (2006); Tung et al. (2020). Items 1 to 4 refer to intinsic motivation, 5 to 7 to extrinsic motivation.
  What is your motivation for becoming a doctor?

  1. ...Sense of calling

2. . . . Personally rewarding work
3. . . . For doing well to others
4. . . . Intellectual curiosity
5. . . . Financial rewards
6. . . . Social status
7. . . . Family tradition

– *Risk attitude*: We used the following four items as used by the MABEL survey Szawlowski et al. (2020): Please indicate your willingness to take risks. . . (1 representing "not at all willing" and 5 "very willing")

1. . . . in general
2. . . . in the financial sector (e.g. investing with uncertain results)
3. . . . professionally (e.g. publicly doubting a colleague)
4. . . . clinically (e.g. recommending controversial treatment)

– *Altruism*: We used the following two questions from Falk et al. (2023)

1. "Are you inclined to donate to a good cause without expecting anything in return?" (1 represents "not at all inclined" and 5 "very inclined")
2. Suppose you unexpectedly received €1,000. How much would you give to charity?

D Online Appendix D. Institutional background

Medical education and access to the medical profession in Italy are highly regulated. The degree course in medicine lasts 6 years. During grades 1-3 students spend most of their time in class and cover the basics of medical theory. During grades 4-6, they combine theoretical classes with medical practice in hospital wards.

In total, 40 Italian universities offer degree courses in medicine - 35 are public and 5 are private. The number of medical school seats available in every university is set every year by a decree of the Ministry of Education after consultations with the universities.

Given severe oversubscription, seats are offered on a competitive ground on the basis of the outcomes of an entry test. The test assesses general competences in logical reasoning, biology, chemistry, physics and math, and is standardized across all public universities, while each private university has its own test. Among public medical schools, the assignment mechanism is akin to a serial dictator. Before taking the test, applicants are asked to rank-order all public medical school and are then assigned to the highest choice with remaining seats, with students scoring higher at the tests being assigned first. Private universities instead admit students on the basis of the ranking at their own test.

According to data for 2019 from Almalaurea, a think-thank collecting information on universities and college-to-work transition, this selective access procedure makes it such that medical students are positively selected with respect to the general population of college students in terms of their family background and are more likely to come from academic high schools (the so-called *licei*).

In order to work as general practitioners (GPs) or specialists, medical graduates need to obtain a specialization degree.[15] Access to specialty courses is restricted and the number of available seats is set by law. Vicarelli and Pavolini (2015) report that a severe oversubscription problem exists also at this level, as the ratio of specialty training places over medical graduates is far below one, and close to 0.7. Access depends on a weighted average of a national test (that covers general topics commonly covered during medical degree courses and is common for both public and private universities) and applicants' CV (the evaluation concerns their final grade at the medical degree, their grade point average, and the quality of their dissertation). The assignment is again similar to a serial dictator, and the best candidates are allocated first. Specialisation courses last between 3 and 5 years, depending on the specialty of choice, and during their specialty training students receive a salary. The training of GPs is instead organized by regions in dedicated schools. As a result, we exclude those students who plan to become GPs from our analysis.

After specialisation, medical graduates start seeking jobs as specialists in private or public hospitals. According to Almalaurea data for 2020, five years after graduation, more than 93% of the medical school graduates are either already working as doctors or enrolled in a specialty course, and less than 1% report to be unemployed.

[15] In addition, until 2020 those medical graduates willing to work as physicians needed to pass a national qualification exam. This exam has then been abolished.

E Robustness tests

Table A5: Baseline WTP-space mixed logit estimates. Excluding respondents listing UK as their favourite foreign European country. Mean and SD.

Variable	Mean	SD
Income (α_i)	0.125***	0.105***
	(0.010)	(0.008)
Job location WTP - reference: Italy		
Favourite foreign EUR country	-13,439***	17,575***
	(1,571)	(1,484)
Professional development opportunities WTP - reference: poor dev. opp.		
Some dev. opp.	6,145***	8,040***
	(1,016)	(1,449)
Good dev. opp.	9,950***	6,638***
	(1,016)	(1,331)
Job security WTP - reference: fix-term,non-renewable job		
Fixed-term job, 50% renew prob.	11,237***	6,523**
	(1,123)	(2,615)
Permanent job	9,467***	12,798***
	(1,591)	(1,171)
Working conditions WTP - reference level: poor		
Good working conditions	13,040***	8,862***
	(977)	(1,153)
Match of skills with job content WTP - reference: higher than needed		
Exactly matched	3,277***	6,071***
	(1,028)	(2,104)
Lower than needed	2,675***	7,438***
	(1,055)	(1,326)
ASC_1	-37,258***	5,169***
	(2,109)	(1,118)
ASC_2	-39,093***	3,988***
	(2,268)	(1,949)
Choices	6,640	
Subjects	415	

Notes: This table reports estimates obtained from Equation 3. Observations are weighted to match the population level statistics of the share of females, age and share of students by macro-regions. Robust standard errors in parentheses. *, p<.10, ** p<.05, *** p<.01.

Table A6: Mean WTP estimates for non-monetary,non-geographical job attributes across jobs located in Italy and in respondents' favourite foreign European country (abroad). Excluding respondents listing UK as their favourite foreign European country.

Variable	Home	Abroad	Difference
Professional development opportunities WTP - reference: poor dev. opp.			
Some dev. opp.	11,988***	-1,823	-13,811***
	(2,480)	(1,889)	(3,497)
Good dev. opp.	9,542***	7,621***	-1,921
	(1,991)	(1,575)	(2,745)
Job security WTP - reference: fix-term,non-renewable job			
Fixed-term job, 50% renew prob.	3,800***	19,840***	16,039***
	(1,725)	(1,459)	(2,245)
Permanent job	13,875***	1,229	-12,647***
	(2,071)	(2,711)	(3,356)
Working conditions WTP - reference level: poor			
Good working conditions	13,284***	12,292***	-992
	(1,509)	(1,552)	(2,059)
Match of skills with job content WTP - reference: higher than needed			
Exactly matched	8,344 ***	-2,394*	-10,738***
	(2,028)	(1,827)	(2,493)
Lower than needed	8,635***	-3,752***	-12,387***
	(1,461)	(1,536)	(2,306)
Choices		6,640	
Subjects		415	

Notes: This table reports estimates obtained from Equation 5. Observations are weighted to match the population level statistics of the share of females, age and share of students by macro-regions. Robust standard errors in parentheses. *, p<.10, ** p<.05, *** p<.01.

Table A7: Baseline unweighted WTP-space mixed logit estimates. Mean and SD.

Variable	Mean	SD
Income (α_i)	0.107***	0.075***
	(0.005)	(0.004)
Job location WTP - reference: Italy		
Favourite foreign EUR country	-13,473***	19,149***
	(996)	(959)
Professional development opportunities WTP - reference: poor dev. opp.		
Some dev. opp.	6,644***	7,291***
	(822)	(1,160)
Good dev. opp.	11,479***	8,330***
	(799)	(827)
Job security WTP - reference: fix-term,non-renewable job		
Fixed-term job, 50% renew prob.	12,435***	3,198
	(727)	(2,855)
Permanent job	10,131***	14,481***
	(917)	(904)
Working conditions WTP - reference level: poor		
Good working conditions	13,113***	8,661***
	(653)	(667)
Match of skills with job content WTP - reference: higher than needed		
Exactly matched	3,852***	6,093***
	(828)	(1,140)
Lower than needed	2,835***	7,060***
	(756)	(849)
ASC_1	-38,952***	5,383***
	(1,112)	(747)
ASC_2	-40,764***	849***
	(1,220)	(2,290)
Choices	8,432	
Subjects	527	

Notes: This table reports estimates obtained from Equation 3. Robust standard errors in parentheses. *, p<.10, ** p<.05, *** p<.01.

Table A8: Unweighted mean WTP estimates for non-monetary,non-geographical job attributes across jobs located in Italy and in respondents' favourite foreign European country (abroad).

Variable	Home	Abroad	Difference
Professional development opportunities WTP - reference: poor dev. opp.			
Some dev. opp.	13,235***	-942	-14,177***
	(1,499)	(1,525)	(2,336)
Good dev. opp.	12,556***	7,338***	-5,217 ***
	(1,477)	(1,430)	(2,376)
Job security WTP - reference: fix-term,non-renewable job			
Fixed-term job, 50% renew prob.	3,821***	22,129***	18,309***
	(1,149)	(1,225)	(1,884)
Permanent job	15,499***	3,113*	-12,387***
	(1,441)	(1,705)	(2,498)
Working conditions WTP - reference level: poor			
Good working conditions	14,207***	11,250***	-2,957
	(1,207)	(1,246)	(2,092)
Match of skills with job content WTP - reference: higher than needed			
Exactly matched	9,082***	-2,055	-11,137***
	(1,327)	(1,306)	(2,014)
Lower than needed	9,439***	-4,714***	-14,153***
	(1,264)	(1,493)	(2,292)
Choices		8,432	
Subjects		527	

Notes: This table reports estimates obtained from Equation 5. Robust standard errors in parentheses. *, p<.10, ** p<.05, *** p<.01.

Table A9: Baseline WTP conditional logit estimates.

Variable	WTP
Job location WTP - reference: Italy	
Favourite foreign EUR country	-10,894***
	(1,046)
Professional development opportunities WTP - reference: poor dev. opp.	
Some dev. opp.	8,947***
	(958)
Good dev. opp.	12,750***
	(1,005)
Job security WTP - reference: fix-term,non-renewable job	
Fixed-term job, 50% renew prob.	13,130***
	(922)
Permanent job	11,161***
	(1,022)
Working conditions WTP - reference level: poor	
Good working conditions	13,898***
	(758)
Match of skills with job content WTP - reference: higher than needed	
Exactly matched	4,843***
	(1,027)
Lower than needed	4,278***
	(987)
ASC_1	-37,609***
	(2,118)
ASC_2	-40,006***
	(2,217)
Choices	8,432
Subjects	527

Notes: Observations are weighted to match the population level statistics of the share of females, age and share of students by macro-regions. Robust standard errors in parentheses. *, p<.10, ** p<.05, *** p<.01.

Table A10: Conditional logit WTP estimates for non-monetary,non-geographical job attributes across jobs located in Italy and in respondents' favourite foreign European country (abroad).

Variable	Home	Abroad	Difference
Professional development opportunities WTP - reference: poor dev. opp.			
Some dev. opp.	17,537***	-2,696	-20,234***
	(1,839)	(1,764)	(2,879)
Good dev. opp.	12,388***	8,068***	-4,319*
	(1,769)	(1,473)	(2,532)
Job security WTP - reference: fix-term,non-renewable job			
Fixed-term job, 50% renew prob.	6,606***	20,645***	14,039***
	(1,598)	(1,460)	(2,361)
Permanent job	20,000***	2,443	-17,557***
	(2,045)	(1,891)	(3,301)
Working conditions WTP - reference level: poor			
Good working conditions	15,901***	11,925***	-3,976
	(1,528)	(1,406)	(2,512)
Match of skills with job content WTP - reference: higher than needed			
Exactly matched	10,014***	-1,062	-11,076***
	(1,656)	(1,359)	(2,256)
Lower than needed	13,911***	-7,124***	-21,035***
	(1,594)	(1,687)	(2,658)
Choices		8,432	
Subjects		527	

Notes: Observations are weighted to match the population level statistics of the share of females, age and share of students by macro-regions. Robust standard errors in parentheses. *, p<.10, ** p<.05, *** p<.01.

F Online Appendix E. Attribute non-attendance

An important assumption behind our results is that respondents consider all the attributes while making their choices between the job offers. There is a growing body of literature that focus on the importance of attribute non-attendance (ANA) - that is, how the results change if some individuals do not consider some of the attributes in the choice sets Scarpa et al. (2009). ANA occurs for two main reasons Heidenreich et al. (2018). First, it can occur when respondents need to simplify complex choice tasks with many different attributes due to limited cognitive capacity Kahneman (1973); Malhotra (1982); Payne and Bettman (2001). This heuristic approach to decision making violates the assumption of continuous preference on which consumer behaviour theory works Campbell et al. (2008). Second, it can occur if respondents consider one or more attributes to be unimportant. ANA that occurs due to the deeming of some attributes to be unimportant represents preferences and is in line with consumer theory Hensher (2006); Ryan et al. (2009). Considering that respondents in our sample are high-ability medical students, it is unlikely that limited cognitive capacity applies to our context. As a result, in what follows we will assume that ANA occurs if respondents deem attributes as non-important and attach them zero value.

The existing studies generally use two different approaches to measure ANA – stated and inferred. Stated ANA methods ask respondents which attributes they did not consider while making their choice decisions. Inferred ANA methods, on the other hand, use econometric methods (usually some variant of a latent class logit model) to estimate ANA strategies probabilistically Hole (2011); Scarpa et al. (2013); Heidenreich et al. (2018). These approaches constrain the coefficients of the attributes that are considered to be non-attended to zero. As our survey does not include state measures of attribute non-attendance, we assess the robustness of our main findings in Table 2 by adopting one of the most widely utilised inferred ANA approaches, pioneered by Scarpa et al. (2009). This approach exploits a latent class logit (LCL) model which assumes that there are C different classes of respondents with one class following a full attendance strategy and each of the other (C-1) classes following some kind of attribute non-attendance strategy. We assume that the income attribute is always attended as it is unlikely that respondents do not consider the salary level of the job contract and also because it is the numeraire attribute essential for calculating WTPs. That leaves 5 attributes and hence a total of 2^5 or 32 attribute non-attendance strategies. Examples of ANA strategies include any one of the attributes (say personal development opportunity) being non-attended, any two being non-attended and so on. We further assume, following Scarpa et al. (2009) that the non-zero attribute coefficients across the C classes are equal.

Mathematically, a utility function is used to represent each ANA strategy for individual i for alternative j at choice situation t:

$$U_{ijt}(\delta_c) = (\beta \cdot \delta_c)' x_{ijt} + \epsilon_{ijt} \quad (1)$$

where β is a vector of marginal utilities or coefficients of the attributes to be estimated. They are constant across the C classes. The vector x_{ijt} represents the attribute levels and ϵ_{ijt} is an extreme value distributed random error term. The vector δ_c contains only ones and zeros and multiplied with the vector β, they represent different ANA strategies.

We ran into maximum log-likelihood convergence problems while running the 32-class (31 ANA

strategies and 1 fully attended strategy) model. We even tried assuming that the working conditions attribute is always attended based on other supporting evidence, bringing down the number of classes in the LCL model to 16. Unfortunately, the model still did not converge. This is not surprising given that we only have 527 respondents in our final sample.

Hence we decided to run several smaller LCL models with each non-attendance strategy separately (always maintaining the full-attendance strategy as one of the classes in each of these LCL models). Specifically, we ran 5 different LCL models:

1. Any one attribute is non-attended (6-class model with one attribute being non-attended in each class and one class with all attributes being attended).

2. Any two attributes are non-attended (11-class model with 10 of the classes assuming any two attributes being non-attended in some combination and one full-attendance class).

3. Any three attributes are non-attended (11-class model with 10 of the classes assuming any three attributes being non-attended in some combination and one full-attendance class).

4. Any four attributes are non-attended (6-class model with 5 of the classes assuming any four attributes being non-attended in some combination and one full-attendance class).

5. Five attributes are non-attended (2-class model with one class assuming all five attributes to be non-attended and the other class assuming full attendance).

Table A11 shows the results from these 5 models. The columns show the average WTPs of each attribute from the above mentioned models and their corresponding probabilities of being non-attended or skipped. For example, in the 1-ANA model, where any one of the attributes are allowed to be non-attended, the average WTP of going abroad is -€13,039 and the probability that the country attribute is skipped is 0.364. The average WTPs are obtained as the non-zero attribute WTP (which is equal across the classes where the attribute is attended) times the total probability that the attribute is attended to (or $1 - the\ probability\ of\ non-attendance$). The non-attendance probabilities are calculated as the sum of the class shares where the attribute is considered to be non-attended. The results show that regardless of the ANA strategies assumed, the WTPs are quite stable across the models and in line with our main results. Country of work seems to be the attribute that is most susceptible to being non-attended with non-attendance probability reaching as high as 70% suggesting that they are highly susceptible to move abroad if amply compensated by other job attributes. Working conditions seems to be the attribute with the lowest non-attendance probabilities across the models suggesting that respondents really care about their workload, much in line with our home-abroad model (see Table 3).

Table A11: Average WTPs by ANA model with corresponding attribute non-attendance probabilities

Attribute	1-ANA WTPs (1)	ANA prob (2)	2-ANA WTPs (3)	ANA prob (4)	3-ANA WTPs (5)	ANA prob (6)	4-ANA WTPs (7)	ANA prob (8)	5-ANA WTPs (9)	ANA prob (10)
Job location WTP - reference: Italy										
Favourite foreign EUR country	-13,039	0.364	-11,378	0.67	-11,545	0.70	-12,906	0.502	-12,408	0.261
Professional development opportunities WTP - reference: poor dev. opp.										
Some dev. opp.	8,053	0.112	6,623	0.265	4,848	0.384	5,151	0.520	8,303	0.261
Good dev. opp.	12,395	0.112	10,603	0.265	9,046	0.384	7,733	0.520	11,429	0.261
Job security WTP - reference: fix-term,non-renewable job										
Fixed-term job	12,557	0.04	11,942	0.232	11,701	0.460	9,158	0.516	11,417	0.261
Permanent job	11,565	0.04	11,483	0.232	12,630	0.460	7,297	0.516	9,669	0.261
Working conditions WTP - reference level: poor										
Good working conditions	13,722	0.26	14,163	0.153	11,999	0.424	11,488	0.429	13,228	0.261
Match of skills with job content WTP - reference: higher than needed										
Exactly matched	4,516	0.20	2,296	0.426	-248	0.575	2,711	0.557	4,837	0.261
Lower than needed	3,347	0.20	2,620	0.426	-124	0.575	3,145	0.557	4,368	0.261

Notes: This table reports estimates obtained from the latent class logit model used to identify attribute non-attendance (ANA) patterns. The columns show the average WTPs of an attribute from the relevant ANA model and its corresponding non-attendance probability.

Supplementary References

ANELLI, M. (2019): "University to Work Transition," *IGIER policy brief.*

CAMPBELL, D., W. G. HUTCHINSON, AND R. SCARPA (2008): "Incorporating Discontinuous Preferences into the Analysis of Discrete Choice Experiments," *Environmental and Resource Economics*, 41, 401–417.

CHEN, G., S. M. GULLY, AND D. EDEN (2001): "Validation of a New General Self-Efficacy Scale," *Organizational Research Methods*, 4, 62–83.

FALK, A., A. BECKER, T. DOHMEN, D. HUFFMAN, AND U. SUNDE (2023): "The preference survey module: A validated instrument for measuring risk, time, and social preferences," *Management Science*, 69, 1935–1950.

HEIDENREICH, S., V. WATSON, M. RYAN, AND E. PHIMISTER (2018): "Decision Heuristic or Preference? Attribute Non-Attendance in Discrete Choice Problems," *Health Economics*, 27, 157–171.

HENSHER, D. A. (2006): "Revealing Differences in Willingness to Pay due to the Dimensionality of Stated Choice Designs: An Initial Assessment," *Environmental and Resource Economics*, 34, 7–44.

HOLE, A. R. (2011): "A Discrete Choice Model with Endogenous Attribute Attendance," *Economics Letters*, 110, 203–205.

KAHNEMAN, D. (1973): *Attention and Effort*, Englewood Cliffs, NJ: Prentice-Hall.

KINGSTON, K. M., C. S. HORROCKS, AND S. HANTON (2006): "Do Multidimensional Intrinsic and Extrinsic Motivation Profiles Discriminate Between Athlete Scholarship Status and Gender?" *European Journal of Sport Science*, 6, 53–63.

LUMPKIN, J. R. (1985): "Validity of a Brief Locus of Control Scale for Survey Research," *Psychological Reports*, 57, 655–659.

MALHOTRA, N. K. (1982): "Information Load and Consumer Decision Making," *Journal of Consumer Research*, 8, 419–430.

NON, A., I. ROHDE, A. DE GRIP, AND T. DOHMEN (2022): "Mission of the Company, Prosocial Attitudes and Job Preferences: A Discrete Choice Experiment," *Labour Economics*, 74, 102087.

OECD (2019): *Health at a glance 2019: OECD indicators*, Paris: OECD Publishing.

PAYNE, J. AND J. BETTMAN (2001): "Preference Choice and Adaptive Strategy Use," in *Bounded Rationality: The Adaptive Toolbox - Dahlem Workshop Reports*, The MIT Press, Cambridge, 103–121.

RYAN, M., V. WATSON, AND V. ENTWISTLE (2009): "Rationalising the 'Irrational': A Think-Aloud Study of Discrete Choice Experiment Responses," *Health Economics*, 18, 321–336.

Ryan, R. M. and E. L. Deci (2000): "Intrinsic and Extrinsic Motivations: Classic Definitions and New Directions," *Contemporary Educational Psychology*, 25, 54–67.

Scarpa, R., T. J. Gilbride, D. Campbell, and D. A. Hensher (2009): "Modelling Attribute Non-Attendance in Choice Experiments for Fural Landscape Valuation," *European Review of Agricultural Economics*, 36, 151–174.

Scarpa, R., R. Zanoli, V. Bruschi, and S. Naspetti (2013): "Inferred and Stated Attribute Non-Attendance in Food Choice Experiments," *American Journal of Agricultural Economics*, 95, 165–180.

Szawlowski, S., B. Harrap, A. Leahy, and A. Scott (2020): "Medicine in Australia: Balancing Employment and Life (MABEL). MABEL User Manual: Wave 11 Release," *Melbourne: Melbourne Institute: Applied Economic and Social Research, The University of Melbourne.*

Tung, Y.-C., Y.-Y. Chou, Y.-H. Chang, and K.-P. Chung (2020): "Association of Intrinsic and Extrinsic Motivating Factors with Physician Burnout and Job Satisfaction: A Nationwide Cross-Sectional Survey in Taiwan," *BMJ open*, 10.

Vicarelli, G. and E. Pavolini (2015): "Health Workforce Governance in Italy," *Health Policy*, 119, 1606–1612.
